# Factors That Affect Large Subunit Ribosomal DNA Amplicon Sequencing Studies of Fungal Communities: Classification Method, Primer Choice, and Error

**DOI:** 10.1371/journal.pone.0035749

**Published:** 2012-04-27

**Authors:** Teresita M. Porter, G. Brian Golding

**Affiliations:** McMaster University, Hamilton, Ontario, Canada; University of California Riverside, United States of America

## Abstract

Nuclear large subunit ribosomal DNA is widely used in fungal phylogenetics and to an increasing extent also amplicon-based environmental sequencing. The relatively short reads produced by next-generation sequencing, however, makes primer choice and sequence error important variables for obtaining accurate taxonomic classifications. In this simulation study we tested the performance of three classification methods: 1) a similarity-based method (BLAST + Metagenomic Analyzer, MEGAN); 2) a composition-based method (Ribosomal Database Project naïve Bayesian classifier, NBC); and, 3) a phylogeny-based method (Statistical Assignment Package, SAP). We also tested the effects of sequence length, primer choice, and sequence error on classification accuracy and perceived community composition. Using a leave-one-out cross validation approach, results for classifications to the genus rank were as follows: BLAST + MEGAN had the lowest error rate and was particularly robust to sequence error; SAP accuracy was highest when long LSU query sequences were classified; and, NBC runs significantly faster than the other tested methods. All methods performed poorly with the shortest 50–100 bp sequences. Increasing simulated sequence error reduced classification accuracy. Community shifts were detected due to sequence error and primer selection even though there was no change in the underlying community composition. Short read datasets from individual primers, as well as pooled datasets, appear to only approximate the true community composition. We hope this work informs investigators of some of the factors that affect the quality and interpretation of their environmental gene surveys.

## Introduction

Nuclear ribosomal DNA (rDNA) markers are widely used in fungal phylogenetic and systematic studies [1]–[4]. In most fungi, rDNA includes the small subunit (SSU, 18S), internal transcribed spacer (ITS, ITS1+5.8S+ITS2), and large subunit (LSU, 25–28S) regions. Though ITS has been proposed as the official fungal ‘barcode’, there are some situations where LSU may be specifically targeted, with or without the adjacent ITS region in amplicon-based environmental sequencing studies [5]–[13].

In contrast with ITS, LSU can be aligned across the diverse range of fungi recovered from environmental samples. The ability to create inclusive alignments means that communities can be analyzed in a phylogenetic context. This approach leverages the observation that closely related taxa often share features such as trophic status in mushroom-forming fungi [14]. In addition to binning sequences by similarity into equally-weighted operational taxonomic units (OTUs), an approach often used with ITS sequences, globally aligned LSU sequences can also be weighted by branch length in a phylogeny. Methods such as the P-test or UniFrac utilize the information content in branch lengths to detect significant differences between communities and to visualize community shifts [15]–[17].

Phylogeny-based community comparison tools can be used with LSU rDNA because it is a mosaic, comprised of both highly variable sequence that provides discriminatory power anchored by highly conserved sequence that can be aligned [18]–[20]. The LSU divergent domains (D), or expansion regions, can show great sequence and length variation among species. An early study described 12 divergent domains responsible for the size increase in the LSU ribosomal RNA (rRNA) gene from prokaryotes to eukaryotes [19]. A more recent study detected 22 variable domains in the eukaryote LSU rRNA gene [20]. Descriptions of these variable regions and secondary structures in eukaryotes have been compared across a range of taxa [19]–[24]. In fungi, an assessment of LSU regions suitable for phylogenetic analysis was conducted, and a suite of primers was developed [18]. Large collections of LSU reference sequences have since been compiled, such as for mushroom-forming fungi [14], [25]. Historically, the D1/D2 region has been used, with or without the corresponding ITS sequence, to identify yeast species [26], [27]. Recently, a 1,500 bp sequence spanning the 3′ SSU+ITS+5′ LSU has been recommended as the barcoding region for arbuscular mycorrhizal fungi [28]. In most fungi, 5′-LSU rDNA is used for genus or higher level taxonomic classifications [29]. Many LSU rDNA sequences are available from GenBank, but additional reference sequences from a broad array of fungi identified by specialists can also be found from the Assembling the Fungal Tree of Life Project (AFTOL) and UNITE databases [2], [30], [31].

This study was prompted by two observations. First, compared with Sanger sequencing, next-generation sequencing (NGS) results in large collections of relatively short reads. This makes primer choice a particularly important variable to target the most informative regions to classify unknown amplicon sequences from environmental sequencing studies. It is currently unknown whether some primer combinations are better than others in terms of LSU classification accuracy. Second, even without cloning, sequence error can still be introduced during mixed-template PCR and NGS [32]–[34]. The extent that this sequence error may affect LSU classification accuracy is unknown. To address these points, we specifically tested the effect of sequence length, primer choice, and sequence error on classification accuracy. We also present a comparison of three automated tools appropriate for use with amplicon-based environmental sequences. The tools we compared are fundamentally different in that they use sequence similarity, sequence composition, or phylogeny as a basis for classification. We hope that this study helps investigators with their experimental design and choose the methods best suited for analyzing their environmental LSU rDNA amplicon sequences.

## Methods

### Mapping primers and variable regions of LSU rDNA

To show the relationship between primers and the variable regions of LSU rDNA we created a map based on the RDN25-1 gene from *Saccharomcyes cerevisiae* GenBank accession NC_001144:455181-451786. We show the 12 divergent domains responsible for the size increase in the large subunit rRNA gene from prokaryotes to eukaryotes [19]. For comparison we also show the 22 variable regions in the eukaryote large subunit rRNA gene [20]. We also mapped the location of primers commonly used in previous environmental sampling studies (Table 1).

**Table 1:** Nuclear large subunit ribosomal DNA (LSU rDNA) primers.

Primer	Sequence (5′ to 3′)	Coordinates with respect to *Saccharomyces cerevisiae* NC_001144.5: 455181 to 451786	Reference
LR0R	ACCCGCTGAACTTAAGC	26–42	Vilgalys lab^1^
LR1^2^	AGCATATCAATAAGCGGAGGA	40–60	[89]
NL-1	GCATATCAATAAGCGGAGGAAAAG	41–64	[90]
LR3R	GTCTTGAAACACGGACC	639–655	Vilgalys lab^1^
NL-4	GGTCCGTGTTTCAAGACGG	655–637	[90]
TW13	GGTCCGTGTTTCAAGACG	655–638	[91]
LR3	GGTCCGTGTTTCAAGAC	655–639	[36]
NDL22^3^	TGGTCCGTGTTTCAAGACG	656–638	[89]
LR16	TTCCACCCAAACACTCG	691–675	[92]
LR5	ATCCTGAGGGAAACTTC	966–950	[36]
nLSU1221R	CTAGATGAACYAACACCTT	1222–1204	[5]
LR7	TACTACCACCAAGATCT	1449–1433	[36]

1 Vilgalys mycology lab http://biology.duke.edu/fungi/mycolab/.

2 Same as LSU 0061 [93].

3 Same as LSU 0599 [93].

### Assembling LSU rDNA datasets

We compiled a well-annotated LSU rDNA sequence set for our simulations using BioPerl (scripts available from T.M. Porter) [35]. We used the following GenBank search terms: Fungi[Organism] AND (“large ribosomal subunit” OR 28S OR 26S OR 25S) NOT (mitochondrial OR mitochondrion OR “uncultured”[TITL] OR “environmental”[TITL] OR “endophyte”[TITL] OR “cf.”[TITL] OR “sp.”[TITL] OR “aff.”[TITL]) AND “AFTOL” [Sept. 21, 2011]. We limited our search to sequences generated by the Assembling the Fungal Tree of Life project (AFTOL) because fungal systematists identified these specimens and classifications represent the current state of taxonomic knowledge. We initially retrieved 1,201 sequences. Results were filtered to retain non-redundant sequences identified to the species level with a minimum sequence length of 100 bp to avoid short partial sequences. This dataset is referred to as the ‘long’ LSU rDNA sequence dataset.

**Figure 1:**
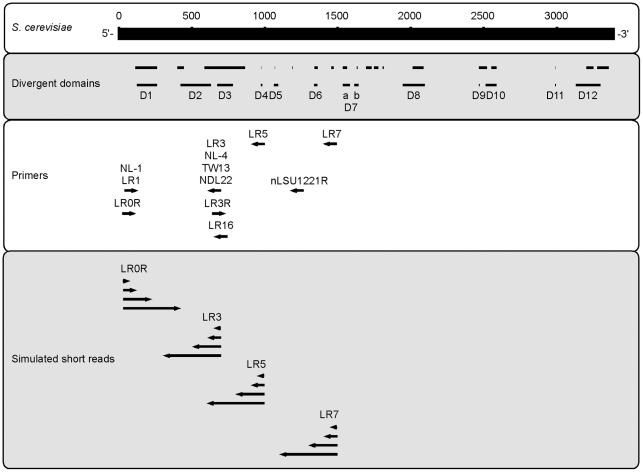
Schematic diagram of large subunit ribosomal DNA (LSU rDNA). In the top frame, the LSU rDNA region for *Saccharomyces cerevisiae* (RDN25-1) NC_001144.5: 455181-451786 is shown. In the second frame, variable sequence regions from Schnare et al. [24] (top) and Hassouna et al. [20] (bottom) have been mapped with respect to the *S. cerevisiae* sequence. In the third frame, the position of some commonly used LSU rDNA primers are shown. In the bottom frame, the position and length of fragments simulated for this study are shown.

To compare the performance of various sub-regions of LSU rDNA for taxonomic assignment, we subsampled this dataset according to what would be obtained using primers that have been previously used in fungal amplicon-based environmental sequencing and span the 5′ LSU rDNA region commonly used in fungal phylogenetics: LR0R, LR3, LR5, and LR7 (Figure 1; Table 1) [36], [37]. Though this represents only a fraction of the primers actually used in previous work, the regions targeted by many primers are similar (Figure 1). We identified the primer binding regions based on sequence similarity using BioPerl scripts allowing up to one mismatch with the primer sequence [35]. For each of these regions, we clipped sequences to various lengths: 50 bp, 100 bp, 200 bp, and 400 bp to simulate the read lengths obtained from current NGS platforms (Figure 1). These are referred to as the ‘simulated short read’ datasets. In mixed template PCR, it is known that primer amplification bias can also have a significant effect on taxonomic recovery [38]; however, we do not specifically simulate this here.

### Sequence Classification

To assess the performance of LSU rDNA sequences for fungal classification we used three methods: 1) BLAST followed by Metagenomic Analyzer parsing (BLAST + MEGAN) [39]–[41]; 2) the Statistical Assignment Package (SAP) [42], [43]; and, 3) the Naive Bayesian Classifier (NBC) available through the Ribosomal Database Project (RDP) website (http://rdp.cme.msu.edu) [44], [45]. Each method classifies sequences to a variety of taxonomic ranks using fundamentally different methods such as local sequence similarity, phylogenetic signal, or sequence composition, respectively. SAP and NBC also provide a measure of confidence that can be used to predict correct taxonomic assignments. The usage details for each method are described below.

For each simulation, we measured recovery, erroneous recovery, and coverage. Recovery was measured as the proportion of queries that were correctly classified. Erroneous recovery was measured as the proportion of queries that were incorrectly classified. Coverage was measured as the total number of (correctly or incorrectly) classified sequences. The proportion of queries that could not be classified is equal to the original number of queries submitted minus coverage. Coverage reflects the differential ability to classify reads to different taxonomic ranks because of incomplete sequence annotations in the GenBank nucleotide database or NBC fungal training set, because of methodological differences during classification, or because of the amount of variation present in the query sequence. We did not account for synonyms or anamorph-teleomorph names because there is no automated way to do this. It is possible that this may contribute to a small number of false negatives during classification.

### Search Scenarios

To simulate searching a complete database, the GenBank accession of the query sequence was left in the database and permitted to be a valid search result; this is referred to as a ‘complete’ database search. To simulate searching a database that is potentially incomplete, we repeated the analyses using a cross-validation (‘leave one out’) search similar to that used by Liu et al. [46]. In the ‘leave one out’ search scenario we excluded the GenBank accession of the query from the search results. In this situation, more than one LSU rDNA sequence per species would be needed in the nucleotide database for a correct classification to the species rank; and more than one sequence per genus would be needed for a correct classification to the genus rank, and so forth. Incorrect classifications would then be due to a lack of sequence variation, misidentified database sequences, or insufficient database coverage. The problem of incomplete reference databases can be a significant barrier with using DNA sequences for taxonomic classification and we wanted to reflect this in our simulations [47]–[50]. To facilitate comparisons among all three methods, we did not enforce any minimum measure of confidence for assignments. However, to see the effect of enforcing a minimum measure of confidence, we repeated some analyses using the default cutoffs recommended by NBC and SAP.

### BLAST + MEGAN: Sequence similarity-based classification

MEGAN parses BLAST reports or NBC classifications and summarizes results at a variety of taxonomic ranks according to the GenBank taxonomy using a Lowest Common Ancestor (LCA) algorithm [39]–[41]. MEGAN has been previously used to classify LSU rDNA/cDNA amplicon sequences produced by NGS [9], [12]. When we use MEGAN to parse BLAST output comprised of local pairwise alignments, the BLAST + MEGAN method can be thought of as a sequence similarity-based approach. We used BLAST 2.2.24+ with the blastn algorithm, default settings, both with and without the ‘-negative_gilist’ option to search a local installation of the GenBank nucleotide database for ‘leave one out’ and ‘complete’ searches [October 2011]. The LCA algorithm assigns taxa to the lowest possible taxonomic rank that presumably reflects the level of sequence variation present in the query sequence compared with reference sequences. The LCA settings we used to parse BLAST reports were minimum support  = 1, minimum score  = 50 (for 50 bp fragments) or 100 (for all other sequence lengths), top percent  = 1.0, and winscore  = 0.0. We disabled all taxa in the NCBI taxonomy that MEGAN uses except for Eukaryotes to try to avoid parsing insufficiently identified sequences from environmental samples. If we were working with field samples, parsing environmental sequences with MEGAN may help to classify reads similar to sequences currently only known from other environmental sequencing studies [49]. We compared the taxonomic lineage of the original dataset with MEGAN classifications using the [R] Bioconductor package ‘genomes’ and custom Perl scripts [51].

### The RDP naïve Bayesian classifier: sequence composition-based taxonomic assignment

NBC uses a naïve Bayesian approach to classify sequences to a variety of taxonomic ranks from domain to genus and provides a confidence estimate for each assignment [44]. Briefly, the bacterial 16S rDNA classifier is a text-based Bayesian classifier that uses a k-mer based approach. The classifier is ‘trained’ using a database of well-identified sequences. The classifier uses the 8 bp oligonucleotide ‘words’ or 8-mers in a query sequence that match words contained in taxa that comprise a genus in the training set to calculate a score. Placement is made to the genus with the highest score. Confidence is estimated using 100 bootstrap replicates. This method is a composition-based method because classifications depend on the k-mer composition of query and reference sequences. Though this tool has been available for bacterial classifications for some time, a fungal LSU rDNA classifier has only recently become available [45]. Their classifier was trained with a 1,400 bp portion of the 5′ end of LSU rDNA from a database of 8,506 sequences. Here, we used the RDP naïve Bayesian rRNA Classifier version 2.2 with fungal LSU training set 1, with and without the recommended confidence thresholds of 50% for sequences less than 250 bp or 80% for longer sequences. We compared the taxonomic lineage of the original dataset with NBC classifications using custom Perl scripts.

### SAP: Phylogeny-based taxonomic assignment

SAP automates the process of conducting BLAST searches, homolog compilation, alignment, and phylogenetic analysis [42], [43]. SAP also provides classifications to a variety of taxonomic ranks providing a statistical measure of confidence for each assignment. This method is a phylogeny-based tool that uses global alignments of similar sequences retrieved by BLAST. Though SAP implements a rigorous Bayesian assignment algorithm, here we only use the faster neighbor joining algorithm since it has been previously shown that results from both methods provided similar classifications with ITS rDNA [52]. We used the ‘NJConstrained’ algorithm with and without the default 95% neighbor joining bootstrap proportion to filter results considered good taxonomic assignments. After testing numerous variations of parameters, we ultimately used the following settings: hits were retained if the local sequence similarity with the query was at least 90%; homologs were compiled that represent at least one phylum, two classes, three orders, five families, ten genera, and one individual per species if possible. We repeated these analyses both with and without the ‘–forceexcludegilist’ option for ‘leave one out’ and ‘complete’ database searches. We compared the taxonomic lineage of the original dataset with SAP classifications using custom Perl scripts.

### Error simulations

There are many points during data generation where sequence errors may be introduced, such as during mixed-template PCR, cloning, and sequencing [32]–[34]. We simulated errors in our data to test classification robustness. We used our original 200 bp short datasets, one from each primer (LR0R, LR3, LR5, and LR7) to represent mock communities. We then created four more mock communities for each primer with varying levels of per-base error rates: 0.01%, 0.1%, 1%, and 10% using a custom Perl script. Classifications were made using BLAST and a ‘leave one out’ approach followed by MEGAN parsing. NBC was used ‘as is’ from the RDP website with the recommended 50% confidence cutoff for fragments shorter than 250 bp. Classifications were summarized to the genus rank. We tracked sequences that were correctly classified with 0% error, and followed their change in recovery as levels of simulated sequence error were increased to 10%. Chimeric sequences are another source of error, however, we did not specifically simulate this. Though not used in this study, LSU rDNA chimera detection from field samples can be performed using UCHIME [53].

We also compared taxonomic composition similarity across mock communities using the comparison tools in MEGAN. Classifications from BLAST and NBC were imported into MEGAN and summarized at the order rank. LCA parameters for processing BLAST reports were as described above. LCA parameters for processing NBC classifications were minimum support  = 1, minimum score  = 50 (recommended for fragments <250 bp), and top percent  = 100. Distance matrices were generated in MEGAN using two ecological indices. The Bray-Curtis statistic quantifies dissimilarity among samples in pairwise comparisons, and has been found to be a robust measure of ecological distance [54], [55]. A phylogeny-based metric, UniFrac, emphasizes the amount of branch length unique to either of two datasets compared with the total amount of branch length in a phylogeny. In environmental sequencing studies, this is interpreted as representing evolution among lineages unique to a site that may reflect adaptation to a specific environment [16]. MEGAN calculates a simplified UniFrac distance based on GenBank taxonomy. The distance matrices calculated by MEGAN were visualized using non-metric multidimensional scaling (NMDS) in R using the ‘ecodist’ package with default settings (2 dimensions, 10 iterations, maximum stress = 1e-12) [56].

To control for variable community sizes, we only analyzed simulated short read sequences (200 bp) generated from the same parent sequence where all four primers could be detected [57]. This resulted in four equally sized datasets (33 taxa each). For comparison, we also analyzed the taxonomic assignments from the parent sequences (average length 3,098 bp) referred to as the reference set and this represents the true community composition (Figure S1). We confirmed that BLAST using a complete database search followed by MEGAN classifications resulted in no classification errors in the parent sequences. Finally, we pooled the simulated short read assignments from four primers to see if the resulting community composition was similar to the true community composition.

## Results

### Taxonomic assignments using ‘long’ LSU rDNA

The taxonomic breakdown and average sequence length of the ‘long’ rDNA dataset is shown in Table 2. The relatively short average lengths for the Ascomycota and Basidiomycota are artifacts of database composition. Unfortunately, most of the fully identified fungal sequences in the GenBank nucleotide database are partial and only 500–700 bp in length (Figure S2, File S1). Complete species names and GenBank accession numbers for the long LSU rDNA dataset are shown in Table S1.

**Table 2:** Taxonomic and sequence length breakdown for the ‘long’ LSU rDNA data set.

Taxonomic group	Number of sequences	Average length (bp)
Ascomycota	447	1341
Basidiomycota	323	1337
Chytridiomycota	22	3154
Kickxellomycotina	7	3690
Mucoromycotina	7	3188
Glomeromycota	5	3241
Blastocladiomycota	4	3264
Entomophthoromycotina	3	3054
Zoopagomycotina	3	3327
Neocallimastigomycota	1	3273
Olpidiaceae	1	3237
Rozella clade	1	3189
Total	824	

**Figure 2:**
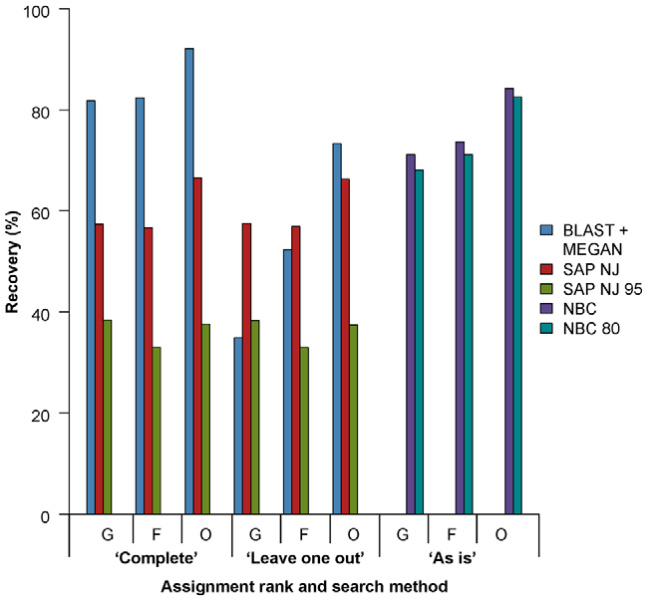
Comparison of methods to classify ‘long’ large subunit ribosomal DNA sequences. Classifications at the genus (G), family (F), and order (O) ranks are shown on the x-axis. Recovery on the y-axis refers to the percentage of queries recovered with a correct classification. Results from BLAST + MEGAN and SAP are directly compared using a ‘complete’ and ‘leave one out’ search scenario. Results from SAP with the default 95% neighbor joining bootstrap cutoff enforced is also shown (SAP NJ 95). Results from NBC run ‘as is’ from the Ribosomal Database Project website are shown separately. Results from NBC with the recommended 80% confidence cutoff are also shown (NBC 80).

We directly compared five classification methods with the ‘long’ LSU rDNA dataset (Figure 2). Using the ‘complete’ search scenario recovery was highest using BLAST + MEGAN. Using the ‘leave one out’ search scenario, SAP with no statistical cutoff performed best for genus and family level assignments, and BLAST + MEGAN performed best for order level assignments. Recovery decreases when the recommended minimum measures of confidence are enforced with NBC and SAP.

### Taxonomic assignments using simulated short reads

**Figure 3:**
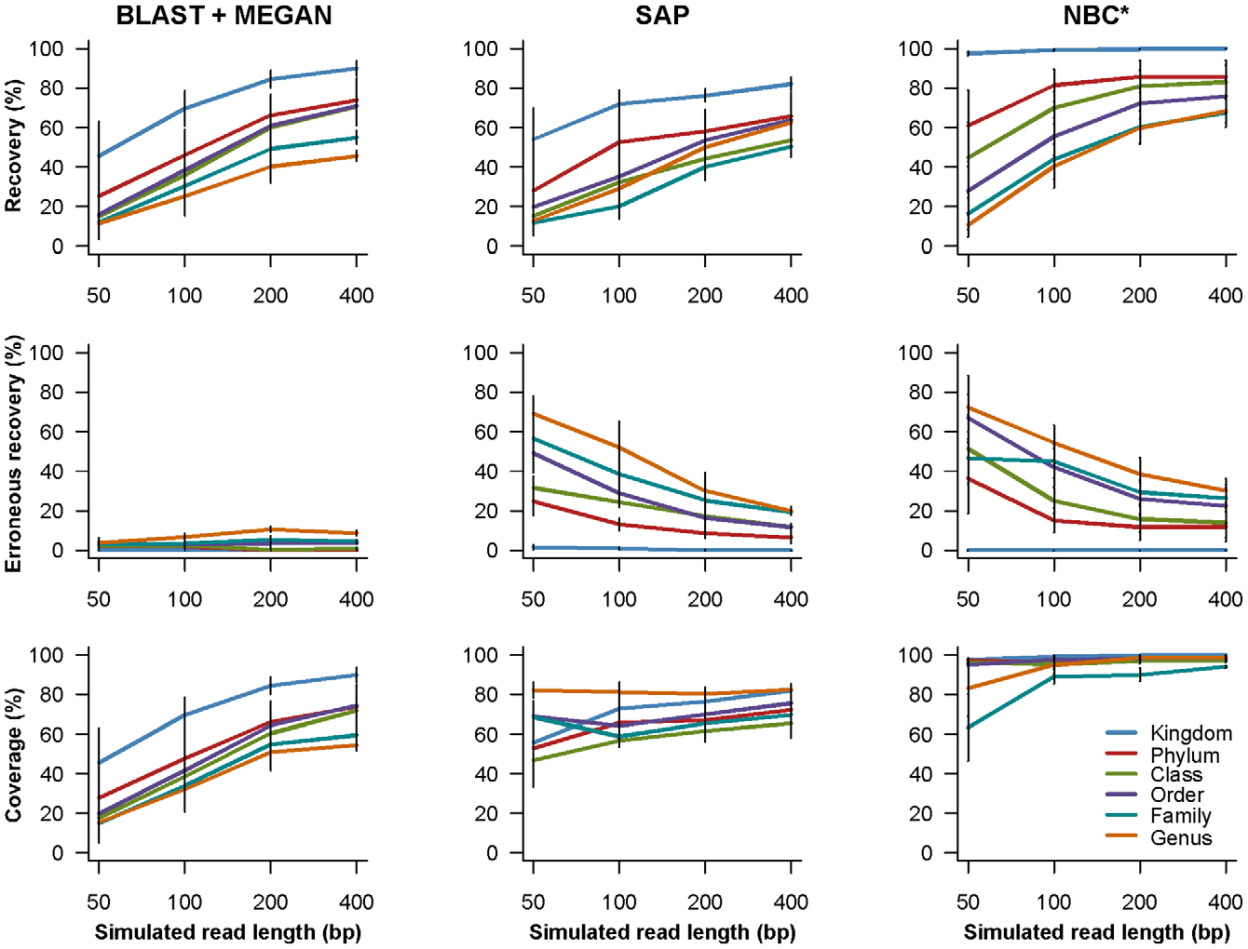
Comparison of classification methods using simulated short read sequences. Simulated read length is shown on the x-axis. In the top row, recovery is shown on the y-axis and refers to the proportion of queries with a correct taxonomic classification. In the middle row, erroneous recovery is shown on the y-axis and refers to the proportion of queries with an incorrect taxonomic classification. In the bottom row, coverage is shown on the y-axis and refers to the proportion of queries for which a classification could be made (correct or incorrect). The results for six taxonomic ranks are shown: kingdom (blue), phylum (red), class (green), order (purple), family (teal), and genus (orange). A ‘leave one out’ search approach was used with BLAST + MEGAN and SAP. The asterisk indicates that NBC was run ‘as is’ from the Ribosomal Database Project website. Bars indicate standard error of the mean using four primers. Statistical cutoffs were not enforced with SAP or NBC to facilitate comparisons with BLAST + MEGAN.

**Figure 4:**
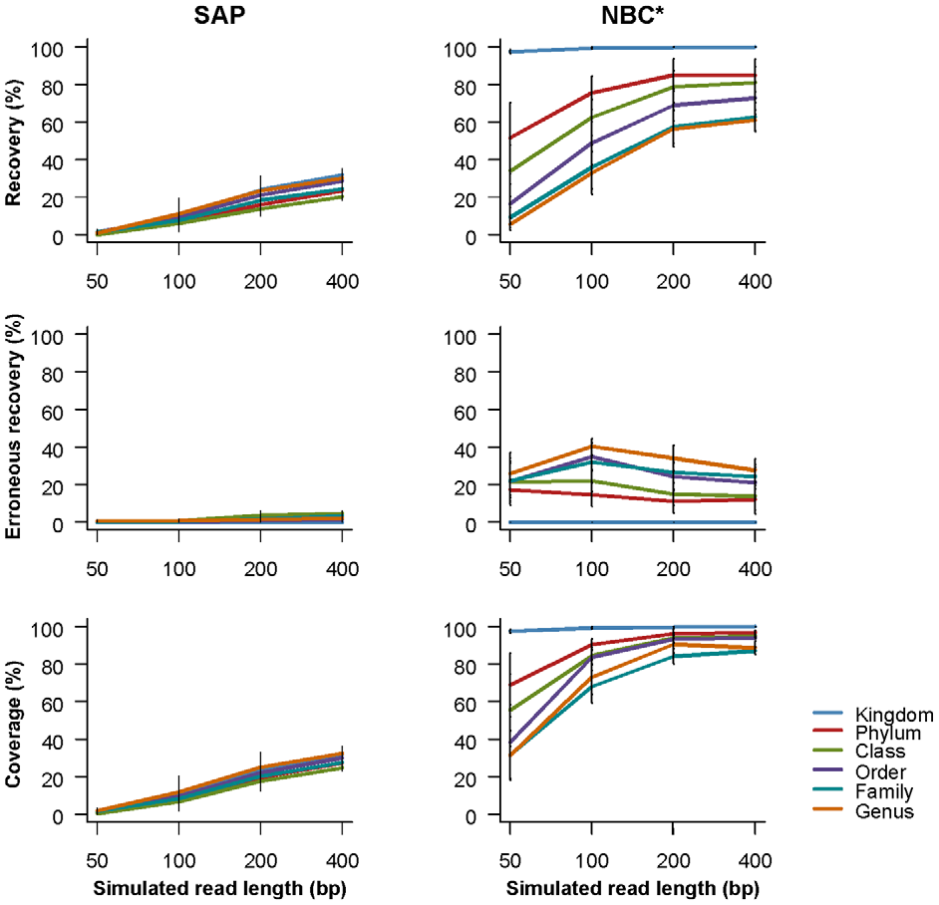
Comparison of classification methods using short read sequences while enforcing a statistical cutoff. Simulated read length is shown on the x-axis. In the top row, recovery is shown on the y-axis and refers to the proportion of queries with a correct taxonomic classification. In the middle row, erroneous recovery is shown on the y-axis and refers to the proportion of queries with an incorrect taxonomic classification. In the bottom row, coverage is shown on the y-axis and refers to the proportion of queries for which a classification could be made (correct or incorrect). The results for six taxonomic ranks are shown: kingdom (blue), phylum (red), class (green), order (purple), family (teal), and genus (orange). A ‘leave one out’ search approach was used with SAP. The asterisk indicates that NBC was run ‘as is’ from the Ribosomal Database Project website. Bars indicate standard error of the mean using four primers. The default statistical cutoffs for SAP (95% neighbor joining bootstrap proportion) and NBC (50% for sequences less than 250 bp, otherwise 80% confidence) are enforced.

The taxonomic breakdown for the ‘simulated short read’ datasets is shown in Table S2. The performance of three classification methods is compared in Figures 3 and 4. Recovery increases with increasing read length for each method. Bars indicate standard error of the mean from four different primers. In Figure 3, BLAST + MEGAN is distinguished by a very low rate of erroneous recovery compared to other methods. In Figure 4, the recommended cutoffs for statistical support are enforced with SAP and NBC. All three measures of SAP performance decrease substantially, indicating that the default statistical cutoff may be too stringent for LSU rDNA. When the NBC default cutoffs are applied, rates of erroneous recovery decrease, especially for the simulated 50 bp reads. Note that the NBC error rate can be even further reduced when NBC results are imported into MEGAN (Figure S3). Corresponding recovery and coverage are only slightly reduced. Because NBC results imported by MEGAN are subject to LCA parsing, any differences in the taxonomy used by GenBank and NBC (family to phylum) result in taxonomic assignments that are collapsed to more inclusive taxonomic ranks.

**Figure 5:**
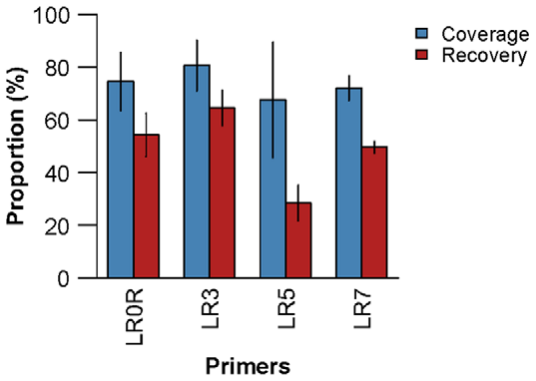
Effect of primer choice on recovery and coverage. Results are shown for 200 bp fragments classified to the genus rank averaged across three methods. We used a ‘leave one out’ approach with BLAST + MEGAN and SAP. NBC was run ‘as is’ from the RDP website. Recovery (blue) and coverage (red) are shown for four primers. Bars indicate standard error of the mean using three classification methods.

Recovery and coverage using four different primers are shown in Figure 5. Results were averaged across the three methods used to create Figure 3. We compared 200 bp sequences classified to the genus rank. Bars indicate standard error of the mean when three classification methods are used. We suggest that differences in recovery and coverage may be due to different levels of sequence variation targeted by the primers. For instance, LR0R and LR3 target sequence in the long D1 and D2 divergent domains whereas LR5 and LR7 target relatively more conserved sequence regions (Figure 1). This is consistent with a previous study that found relatively high levels of pairwise sequence divergence in the D1–D3 regions of 5′ LSU rDNA [18].

### Taxonomic assignments after simulating sequence error

**Figure 6:**
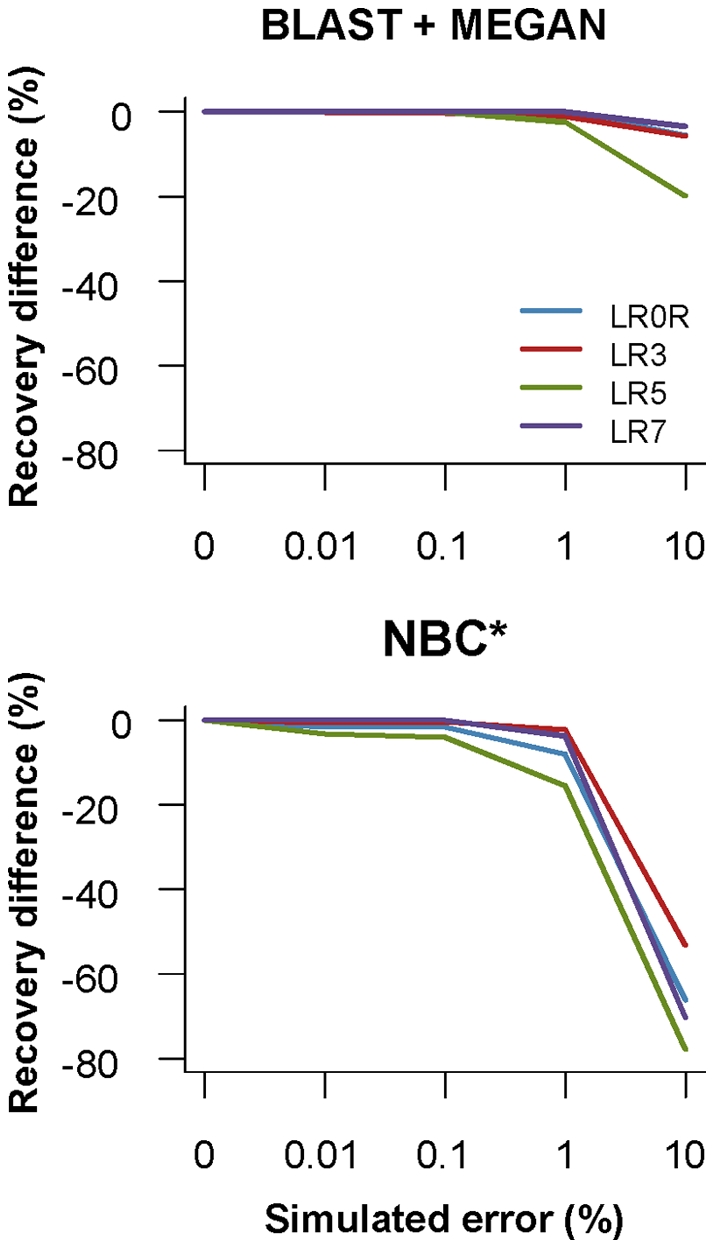
Effect of simulated errors on recovery. Results are shown for 200 bp fragments classified to the genus rank for four primers: LROR (blue), LR3 (red), LR5 (green), and LR7 (purple). BLAST + MEGAN was run with a ‘leave one out’ BLAST search and the asterisk indicates that NBC was run ‘as is’ from the Ribosomal Database Project website. Simulated per-base error rates are shown on the x-axis. Recovery differences compared with correctly classified taxa from the original 200 bp datasets (0% error) are shown on the y-axis.

Recovery at several levels of simulated sequence error is shown in Figure 6. Recovery decreased starting at about 0.1% to 1% simulated error using BLAST + MEGAN. Recovery decreased starting at 0.01% simulated error using NBC. The composition-based classification method, NBC, appears to be more sensitive to sequence error compared with the similarity-based method, BLAST + MEGAN. This may be because in a k-mer based method, any single error in a sequence is propagated into ‘k’ number of words used for classification.

### Effect of primer choice and sequence error on community comparisons

**Figure 7:**
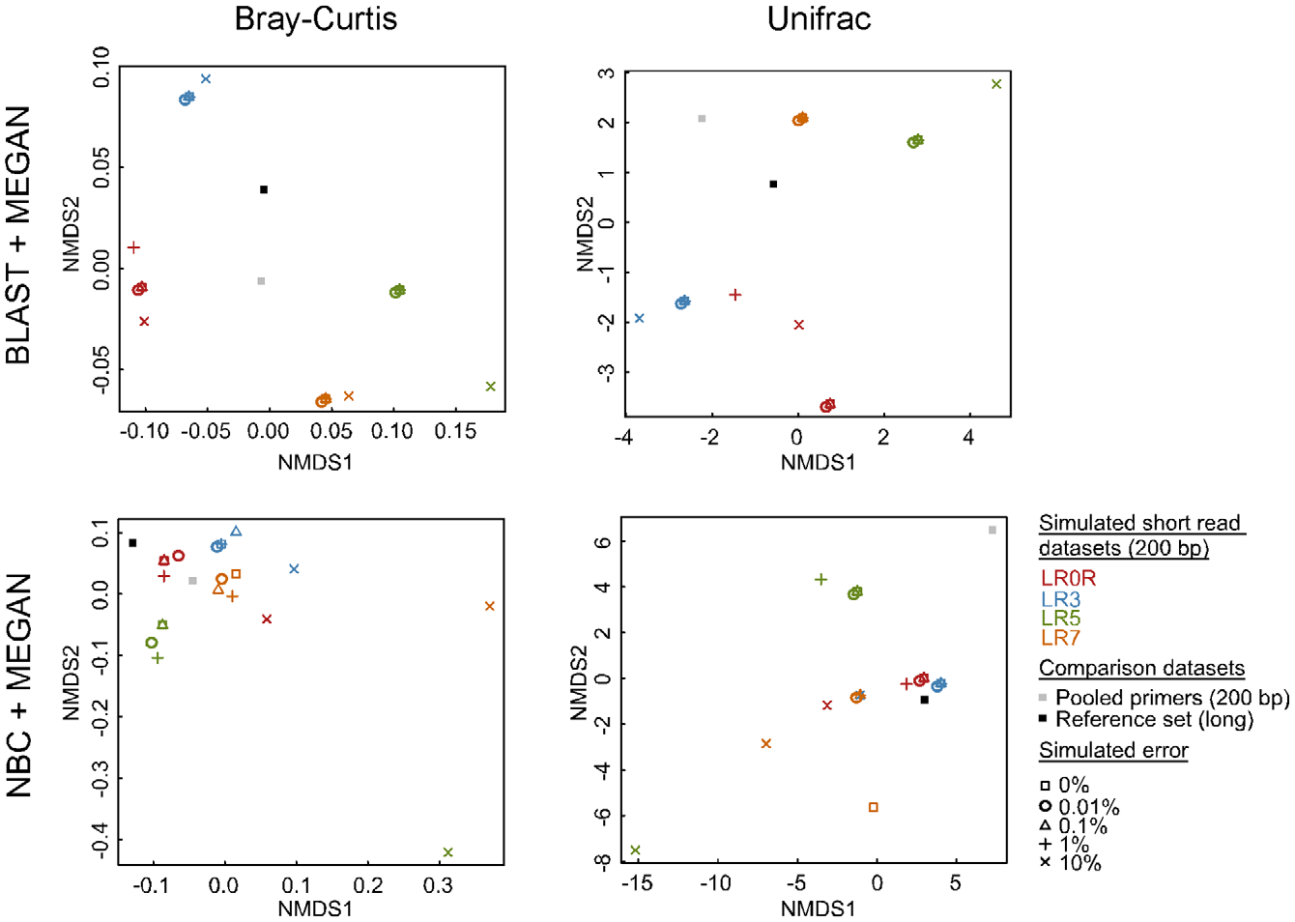
Comparison of simulated communities using non-metric multidimensional scaling. The ‘reference set’ (black square) was comprised of ‘long’ large subunit ribosomal DNA sequences (about 3,000 bp average length) that were classified using MEGAN + BLAST against a complete database or classifications from NBC run ‘as is’ from the Ribosomal Database Project website and imported into MEGAN (NBC + MEGAN). Four mock communities comprised of 200 bp sequences were generated from four primers: LR0R (red), LR3 (blue), LR5 (green), and LR7 (orange). Communities were subjected to per-base error rates of 0% (square), 0.01% (circle), 0.1% (triangle), 1% (+), and 10% (×). Classifications were summarized at the order rank. Similarity of taxonomic composition was compared using Bray-Curtis dissimilarity and a simplified UniFrac measure in MEGAN.

At the cost of reduced specificity, we chose to summarize classifications used in Figure 7 at the order rank to minimize the effect of misidentified or misclassified taxa on taxonomic community comparisons (Figure S4). As expected, we found that each primer individually detects most order-level lineages truly present in the parent community. Even after summarizing classifications at the order rank, we observed community shifts caused by the differential detection of lineages by each primer even though there was no change in the underlying community composition. For example, an order-level lineage, the Leucosporidiales, was detected by the LR7 primer although it was not present in the original parent community.

When BLAST + MEGAN classification was used, the greatest observed community shifts were due to primer choice. This is consistent with the differential recovery we observed among the tested primers (Figure 5). Observed community shifts due to primer selection are reduced with NBC + MEGAN classification where sequence error appears to have a larger effect. This correlates with NBC's increased sensitivity to error shown in Figure 6. Using either classification method, simulated short read primer datasets only approximate the true taxonomic composition.

When data from multiple primers for the same marker are available, the question of whether to pool the data becomes relevant. We observed that the relative configuration of points using two different ecological measures differ, especially for the pooled primer dataset. The Bray-Curtis statistic quantifies dissimilarity among sites regardless of their taxonomic composition. The resulting pooled dataset point falls nearly midway between the four contributing primer datasets. The simplified UniFrac metric implemented in MEGAN, however, measures the proportion of unique branch lengths among datasets. Compared with the pooled dataset, each primer differentially detects lineages represented by varying amounts of branch length (Figure S4). The result is that the pooled dataset falls outside the cluster formed by the four contributing primer datasets. Using either ecological measure, our pooled primer datasets only approximate the true community composition. We suggest that when working with field data, identifying community shifts among ecologically distinct sites may be easier to visualize when data from multiple primers are pooled into a single point.

## Discussion

### Trends in species assignment

Species assignment methods fall into several broad categories. First, similarity-based methods, such as BLAST, are commonly used for amplicon-based environmental sequence classification. It has been observed, however, that the top BLAST hit may not necessarily be the closest phylogenetic neighbor [58]. Additionally, BLAST alone does not automatically make classifications to higher taxonomic ranks where the accuracy for an assignment may be higher. Neither BLAST nor MEGAN provides any measure of confidence for a classification. However, it has been previously shown with ITS rDNA, and in the current study with LSU rDNA, that MEGAN has lower erroneous recovery rates than BLAST, SAP, or NBC [52]. There is a new method developed for classifying pyrosequencing reads using BLAST that does calculate a corresponding probability that the top hit is correct, and this measure would add value to classifications based on BLAST [59].

Second, phylogeny-based classification methods are available such as SAP, pplacer, and the Evolutionary Placement Algorithm (EPA) [42], [43], [60], [61]. These methods use a variety of phylogenetic frameworks such as neighbor joining, maximum likelihood, and Bayesian analysis. Unless SAP can be run in parallel, this method may be best suited for small datasets, because even with the faster neighbor joining algorithm, the BLAST searches, homolog compilation, alignment, and tree-building steps necessary to classify each individual query can be relatively time consuming. A previous study showed that SAP recovery with ITS rDNA was more sensitive to query length than other methods [52]. The current study with LSU rDNA, however, showed that each of the tested methods is similarly sensitive to query length. EPA and pplacer were developed to classify reads from amplicon-based environmental sequencing. They can implement a variety of nucleotide substitution models, and are faster because they use a pre-existing alignment to place unknown sequences onto a reference tree. These particular methods are perhaps best suited for bacterial 16S rDNA classifications because extensive alignments are already publically available [31], [62], [63]. The ARB project provides tools so that new data can be integrated with large sets of pre-aligned sequences facilitating alignment and phylogenetic analyses with a graphical user interphase. The SILVA database does provide a high quality reference LSU alignment (n = 1278, ≥1900 bp) that can be downloaded and used with ARB; however, the hand-curated dataset used to train the RDP fungal LSU classifier is more extensive (n = 8506, 1400 bp) [31], [64]. Though the RDP does support an LSU classifier and a library comparison tool, LSU alignment downloads are not currently available.

Third, composition-based methods are available such as naïve Bayesian classifiers [44], [65]. In this study, we show that the number of sequences classified per minute with NBC far exceeds that using MEGAN or SAP (Figure S5). When processing thousands of OTUs, the difference in run-time can be from hours to days for BLAST-based methods (such as BLAST + MEGAN and SAP) compared with minutes for the composition-based NBC. Additionally, as the reference set of sequences used to train the classifier increases, so too should the number of accurate of classifications. For composition-based methods, a ‘detector’ has recently been developed that improves the performance of a naïve Bayesian classifier by flagging query sequences with no match in the reference set [66]. As shown in this study and elsewhere, the accuracy of nearly all sequence classification methods depends on query length. One newly developed method addresses this problem using a k-mer based approach and mixture modeling to be sequence length independent [67]. This method is currently only available for prokaryote classifications.

In this study, we directly compared classification performance using both complete and incomplete reference databases to highlight that this can be a major limitation in the taxonomic assignment process. BLAST + MEGAN recovery was substantially decreased when working with an incomplete reference database, and SAP performed best with long LSU rDNA sequences. A previous study showed dramatic recovery decreases, with BLAST, BLAST + MEGAN, and SAP when using an incomplete reference database for ITS rDNA sequence classification [52]. Database properties that affect classification performance include breadth and depth of taxonomic representation, classification accuracy of submitted sequences, as well as underlying sequence quality and length. Incomplete databases are due to: fungal diversity in herbaria not represented by sequences in GenBank [47], [48]; insufficiently identified environmental sequences representing newly discovered fungal lineages that are widespread but not readily cultivable using standard methods [5], [49], [50], [68]–[73]; within-individual and within-species rDNA sequence diversity that is not represented in GenBank [74]–[76]; the lack of an ‘official’ fungal barcode [11]; and the sheer diversity of fungal species that need to be accounted for [77], [78]. Even the most advanced taxonomic assignment method can only be as good as the reference database upon which classifications rely.

### The effect of sequencing error on perceived community diversity

Sequence error, such as that generated during PCR, cloning, or sequencing, is a source of noise that can affect the accuracy of classifications and community comparisons. In amplicon-based sequencing studies that use OTU-based estimates of richness, errors can also inflate measurements of alpha diversity such as richness or estimated richness [34], [79]. We show here that error rates exceeding 0.01–1% can begin to affect the recovery of correct classifications. Additionally, we show that sequence error can cause community shifts unrelated to any change in the underlying simulated community. Since errors may vary according to PCR conditions and NGS platform, programs that compensate for this noise need to be used to ensure read quality [80]–[82]. Additionally, clustering reads by at least 1% sequence similarity can reveal singleton sequences that tend to contain many sequence errors [32], [34], [76], [83], [84]. In this study we showed that NBC, a k-mer based taxonomic assignment method, is sensitive to sequence error making de-noising and/or sequence clustering steps particularly important when processing field data.

### Variable performance of LSU rDNA primers

Though there is a history of using LSU rDNA in fungal phylogenetic systematics and amplicon-based environmental sequencing, this is the first simulation study to directly assess the performance of the LSU rDNA marker (Figure S6, File S1). Based on our simulations, we found that the LR0R and LR3 primers targeting the variable D1 and D2 domains yield the highest rate of correct taxonomic assignments. Despite this, ease of PCR amplification will likely dictate which primer sets are the most useful in field studies. Because our study focused on the 5′ LSU rDNA region, our observations do not necessarily reflect the performance of divergent domains in the 3′ LSU rDNA region for taxonomic assignment. In fact, previous studies have shown that D2 in the 5′ LSU rDNA region and D8 in the 3′ LSU rDNA region show the largest size expansions and most sequence divergence [18], [19]. One potential concern with the LR5 primer is that it may amplify a group I intron, though in this study we only detected one taxon with an intron at this position. The presence of group I introns are known to differ between and within species; additionally, they may also be acquired by horizontal transfer [85], [86]. As a result, group I introns do not necessarily share the same evolutionary history as the host genome [87]. Thus care must be taken when sequencing from the LR5 primer.

### Variable performance of classification methods

Compared with the other tested methods, MEGAN produces the lowest error rates. Error rates from BLAST + MEGAN is reduced because the LCA algorithm can reconcile taxonomic assignments to higher ranks when top BLAST hits have heterogeneous taxonomic lineages. This reduces coverage and erroneous recovery at more specific ranks and increases the number of assignments at more inclusive ranks. In some cases, the LCA algorithm will not make an assignment at all, further reducing the rate of incorrect assignments. Although confidence scores produced by SAP and NBC can be used to help predict correct assignments, their error rates are still higher than MEGAN with our dataset. With NBC, this can be partially explained by variations in the taxonomy (family to phylum) used in the fungal training set compared with GenBank. Nevertheless, genus level assignments are still directly comparable so we provide an example illustrating how erroneous assignments may arise. With NBC, so long as the query sequence contains the minimum number of required k-mers, an assignment and confidence value will always be provided. Even if the query genus is not present in the fungal training set, an assignment is still made to the genus with the highest rank-order likelihood score. If this genus assignment happens to be consistent among bootstrap replicates, although it is erroneous, the confidence score will be high resulting in an incorrect assignment supported by a high confidence value. NBC error rates can be reduced by enforcing statistical cutoffs and by importing NBC results into MEGAN though at the expense of slightly reducing recovery, coverage, and taxonomic specificity (Figure S3).

With SAP, enforcing the default statistical cutoff to reduce error rates also drastically reduces recovery and coverage compared with not enforcing any cutoff at all (Figure 3, 4). This reflects the instability of many assignments in bootstrap replicates where characters are re-sampled with replacement. With short query sequences (≤400 bp) and the default cutoff, SAP is outperformed by BLAST + MEGAN (lower error rates) and NBC with the default cutoffs (similar error rates but higher recovery). SAP performs best with long LSU rDNA sequences (∼3000 bp) and no statistical cutoff enforced for genus and family rank assignments (Figure 2).

### Conclusions

For rapid fungal LSU rDNA taxonomic assessments we recommend the use of the Ribosomal Database Project naïve Bayesian classifier (NBC). However, if the chance of erroneous assignments needs to be particularly minimized, then we recommend MEGAN LCA processing of BLAST or NBC results. When NBC results are imported into MEGAN, sample comparisons using multiple methods can be reached very quickly. If the query sequences are long (>400 bp) and processing time is not a pressing issue, then SAP without enforcing any statistical cutoff may be a good alternative. In all cases, summarizing assignments to broader taxonomic ranks can increase the rate of accurate assignments and reduce the error rate, though at the expense of reduced specificity.

Simulation studies can help to evaluate the most appropriate methods for analyzing amplicon-based environmental sequencing data [38], [57], [88]. We presented results from a cross section of classification methods as well as the impact of read length, primer selection, and sequence error on classification accuracy and community composition. We hope this work informs investigators of some of the factors that affect the quality and interpretation of their environmental gene surveys.

## Supporting Information

Figure S1 **Taxonomic composition of the sequences used for non-metric multidimensional scaling community comparisons.** Automated classification of ‘long’ large subunit ribosomal DNA sequences from 33 parent sequences using BLAST against a complete database and MEGAN parsing is shown. This dataset is the ‘reference set’ in Figure 7. All assignments to the species level were verified to be correct. In two cases, MEGAN assigned sequences to higher taxonomic ranks so arrows indicate the species name of the parent sequence.
(PDF)

Figure S2 **Sequence length frequency distribution of fungal ribosomal DNA (rDNA) sequences identified to the species level in GenBank.** Length frequencies for large subunit rDNA (LSU) (black) and the internal transcribed spacer region (ITS) (white) are shown. The number of sequences (y-axis) in each 100 bp bin (x-axis) is shown.
(PDF)

Figure S3 **Comparison of NBC classifications using simulated short read sequences.** Simulated read length is shown on the x-axis. In the top row, recovery is shown on the y-axis and refers to the proportion of queries with a correct taxonomic classification. In the middle row, erroneous recovery is shown on the y-axis and refers to the proportion of queries with an incorrect taxonomic classification. In the bottom row, coverage is shown on the y-axis and refers to the proportion of queries for which a classification could be made (correct or incorrect). The results for six taxonomic ranks are shown: kingdom (blue), phylum (red), class (green), order (purple), family (teal), and genus (orange). NBC was run ‘as is’ from the Ribosomal Database Project website. Bars indicate the standard error of the mean using four primers. In the first column, no statistical cutoffs were enforced. In the second column, the default statistical cutoffs for NBC (50% for sequences less than 250 bp, otherwise 80% confidence) were enforced. In the third column, NBC results were imported into MEGAN using the following LCA parameters: minimum support  = 1, minimum score  = 50 (or 80 for sequences longer than 250 bp), and top percent  = 100.
(PDF)

Figure S4 **Taxonomic breakdown of non-metric multidimensional scaling community comparisons.** Dataset sizes were normalized in MEGAN and taxonomic assignments of 200 bp sequences generated by four primers are compared with the reference set from Figure S1. In part (a), results are summarized at the species rank. In part (b), results are summarized at the order rank. In part (c), results are summarized at the order rank showing results for each primer and associated branch lengths using MEGAN. In each figure, boxes represent the relative number of sequences classified at each node/leaf and colors match those used in Figure 7 for each dataset (0% error).
(PDF)

Figure S5 **Number of classifications per minute.** The average number of classifications per minute is shown for three methods. Bars indicate standard error of the mean using four different primers. For NBC, analysis times for all of our datasets was less than one minute. For BLAST + MEGAN, only the time to conduct local BLAST searches using a single processor was calculated, since MEGAN parsing with our data took less than a minute. With BLAST, the number of classifications per minute could be improved by using multiple processors for each search. For SAP, the total analysis time includes BLAST searches, homolog compilation, alignment, and neighbor joining analyses.
(PDF)

Figure S6 **Articles indexed by Web of Knowledge from 1990–2010.** Research articles with the topic of ITS (white) or LSU (black) phylogenetic systematics and/or barcoding are shown.
(PDF)

Table S1 (DOC)

Table S2 (DOC)
