# Correction: Factors That Affect Large Subunit Ribosomal DNA Amplicon Sequencing Studies of Fungal Communities: Classification Method, Primer Choice, and Error

**DOI:** 10.1371/annotation/188edbe7-9f2e-4031-9155-d4a9337e6257

**Published:** 2012-08-07

**Authors:** Teresita M. Porter, G. Brian Golding

The following information was missing from the Funding section: This project was also funded by the Government of Canada through Genome Canada and the Ontario Genomics Institute through the Biomonitoring 2.0 project (OGI-050). 

