# Supplementary figures and images for: Factors That Affect Large Subunit Ribosomal DNA Amplicon Sequencing Studies of Fungal Communities: Classification Method, Primer Choice, and Error
Source: PLoS One. 2012 Apr 27;7(4):e35749. doi: 10.1371/journal.pone.0035749 (PMC3338786; doi:10.1371/journal.pone.0035749)

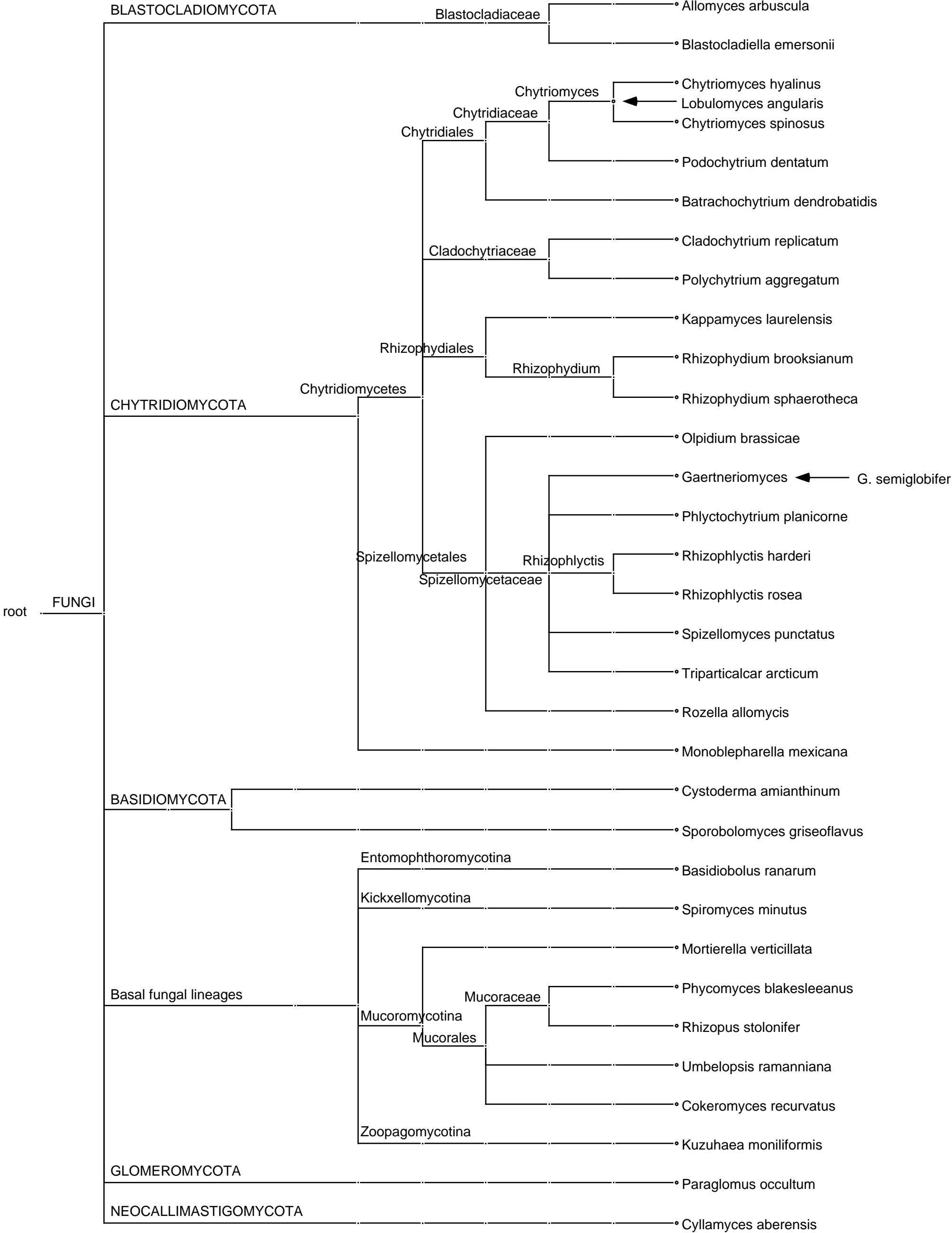

Supplement: Figure S1 — Taxonomic composition of the sequences used for non-metric multidimensional scaling community comparisons. Automated classification of ‘long’ large subunit ribosomal DNA sequences from 33 parent sequences using BLAST against a complete database and MEGAN parsing is shown. This dataset is the ‘reference set’ in Figure 7. All assignments to the species level were verified to be correct. In two cases, MEGAN assigned sequences to higher taxonomic ranks so arrows indicate the species name of the parent sequence. (PDF) [file pone.0035749.s001.pdf]

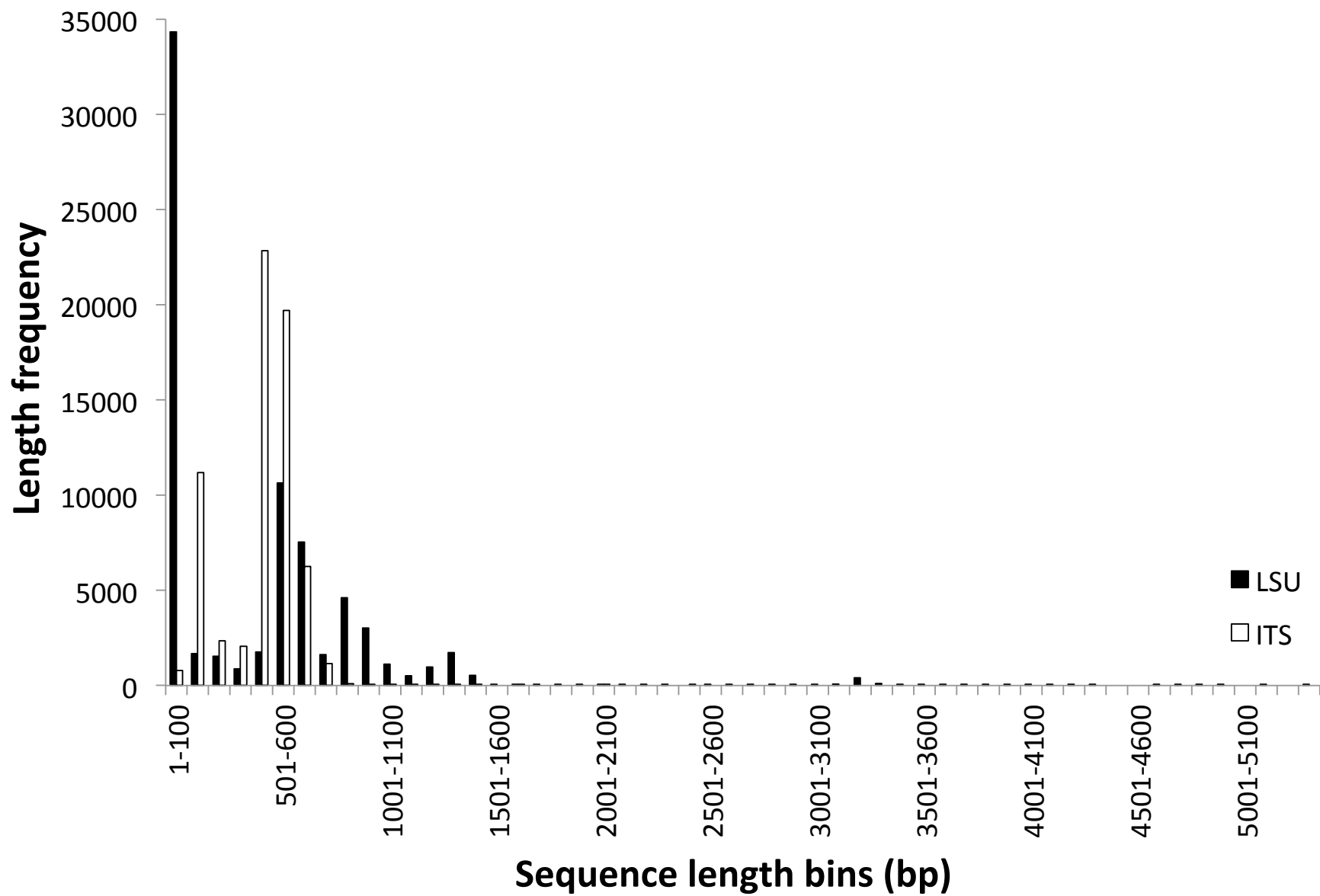

Supplement: Figure S2 — Sequence length frequency distribution of fungal ribosomal DNA (rDNA) sequences identified to the species level in GenBank. Length frequencies for large subunit rDNA (LSU) (black) and the internal transcribed spacer region (ITS) (white) are shown. The number of sequences (y-axis) in each 100 bp bin (x-axis) is shown. (PDF) [file pone.0035749.s002.pdf]

NBC No Cutoff

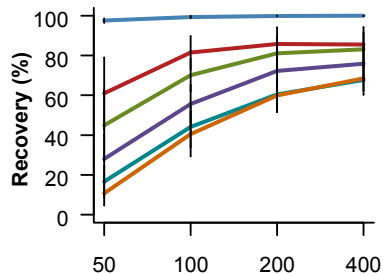

NBC Cutoff

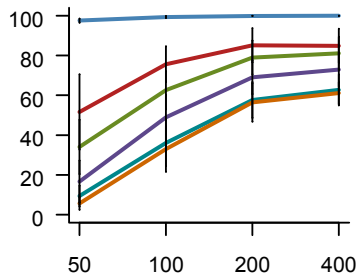

NBC + MEGAN Cutoff

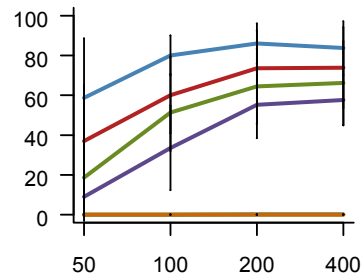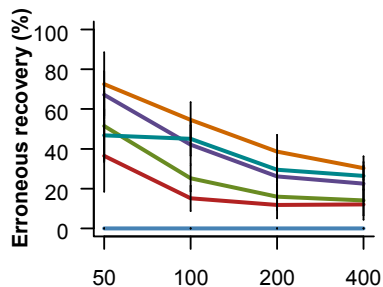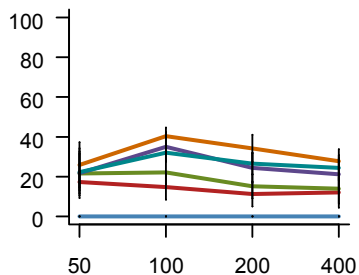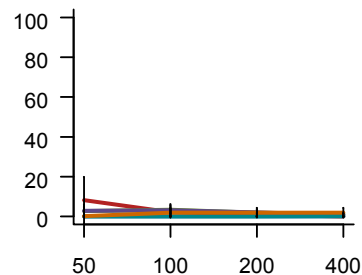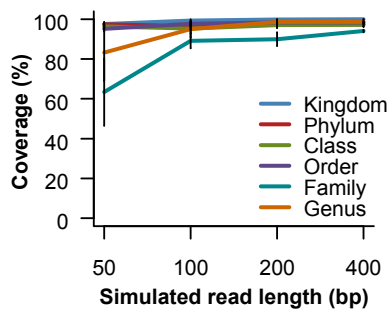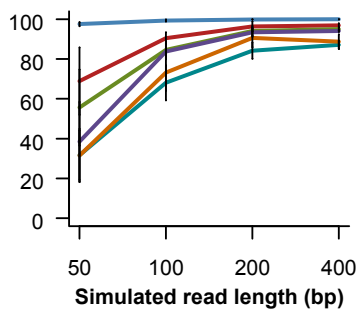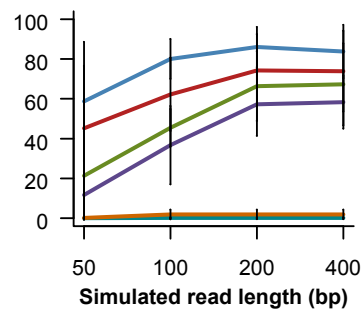

Supplement: Figure S3 — Comparison of NBC classifications using simulated short read sequences. Simulated read length is shown on the x-axis. In the top row, recovery is shown on the y-axis and refers to the proportion of queries with a correct taxonomic classification. In the middle row, erroneous recovery is shown on the y-axis and refers to the proportion of queries with an incorrect taxonomic classification. In the bottom row, coverage is shown on the y-axis and refers to the proportion of queries for which a classification could be made (correct or incorrect). The results for six taxonomic ranks are shown: kingdom (blue), phylum (red), class (green), order (purple), family (teal), and genus (orange). NBC was run ‘as is’ from the Ribosomal Database Project website. Bars indicate the standard error of the mean using four primers. In the first column, no statistical cutoffs were enforced. In the second column, the default statistical cutoffs for NBC (50% for sequences less than 250 bp, otherwise 80% confidence) were enforced. In the third column, NBC results were imported into MEGAN using the following LCA parameters: minimum support = 1, minimum score = 50 (or 80 for sequences longer than 250 bp), and top percent = 100. (PDF) [file pone.0035749.s003.pdf]

a)

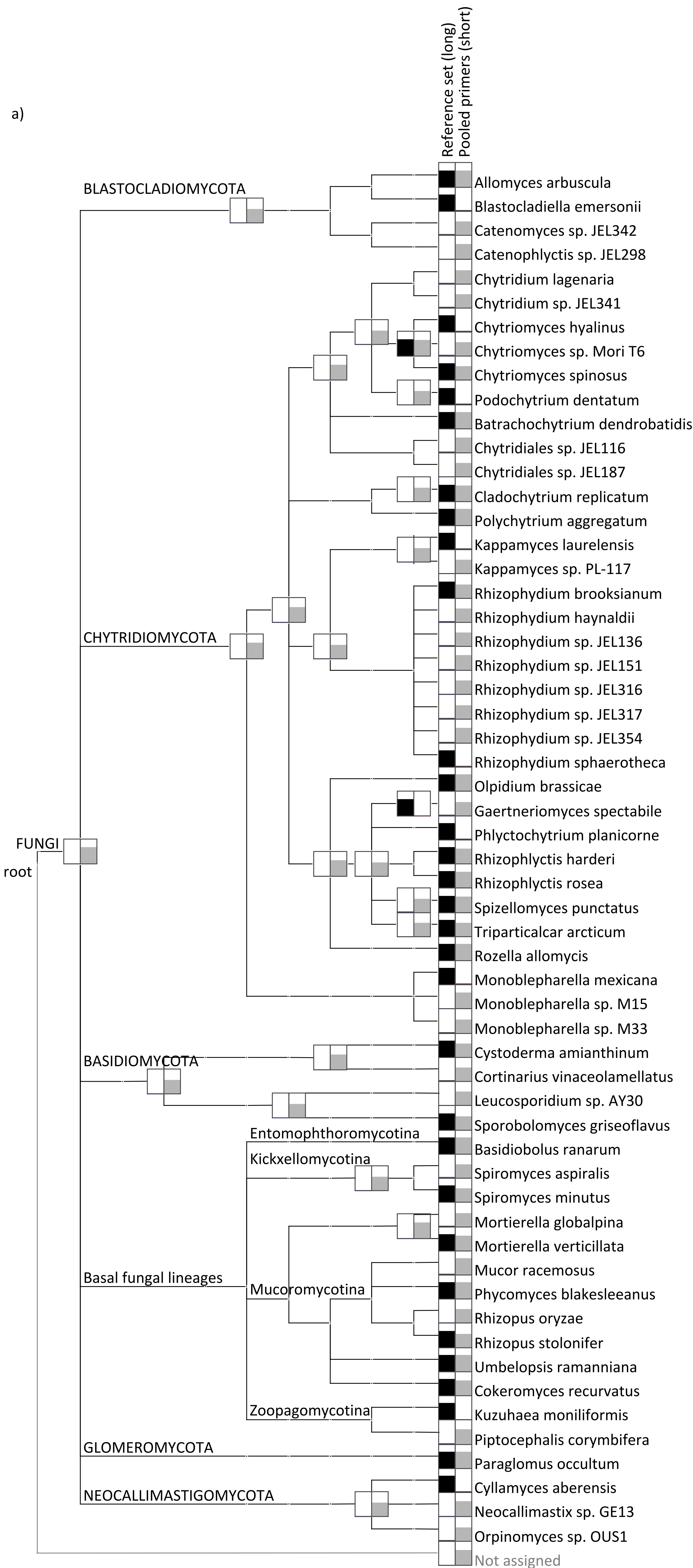

b)

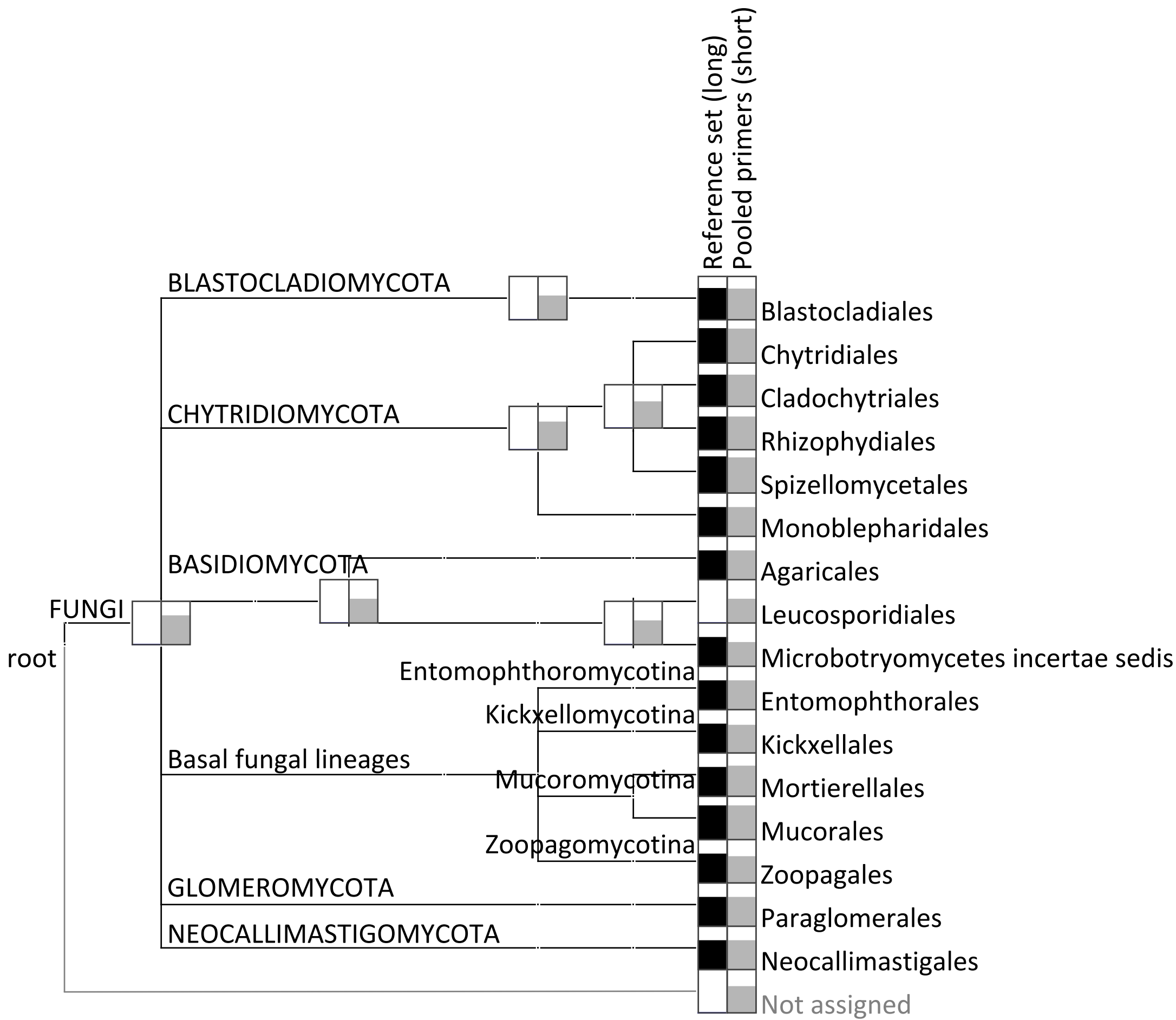

c)

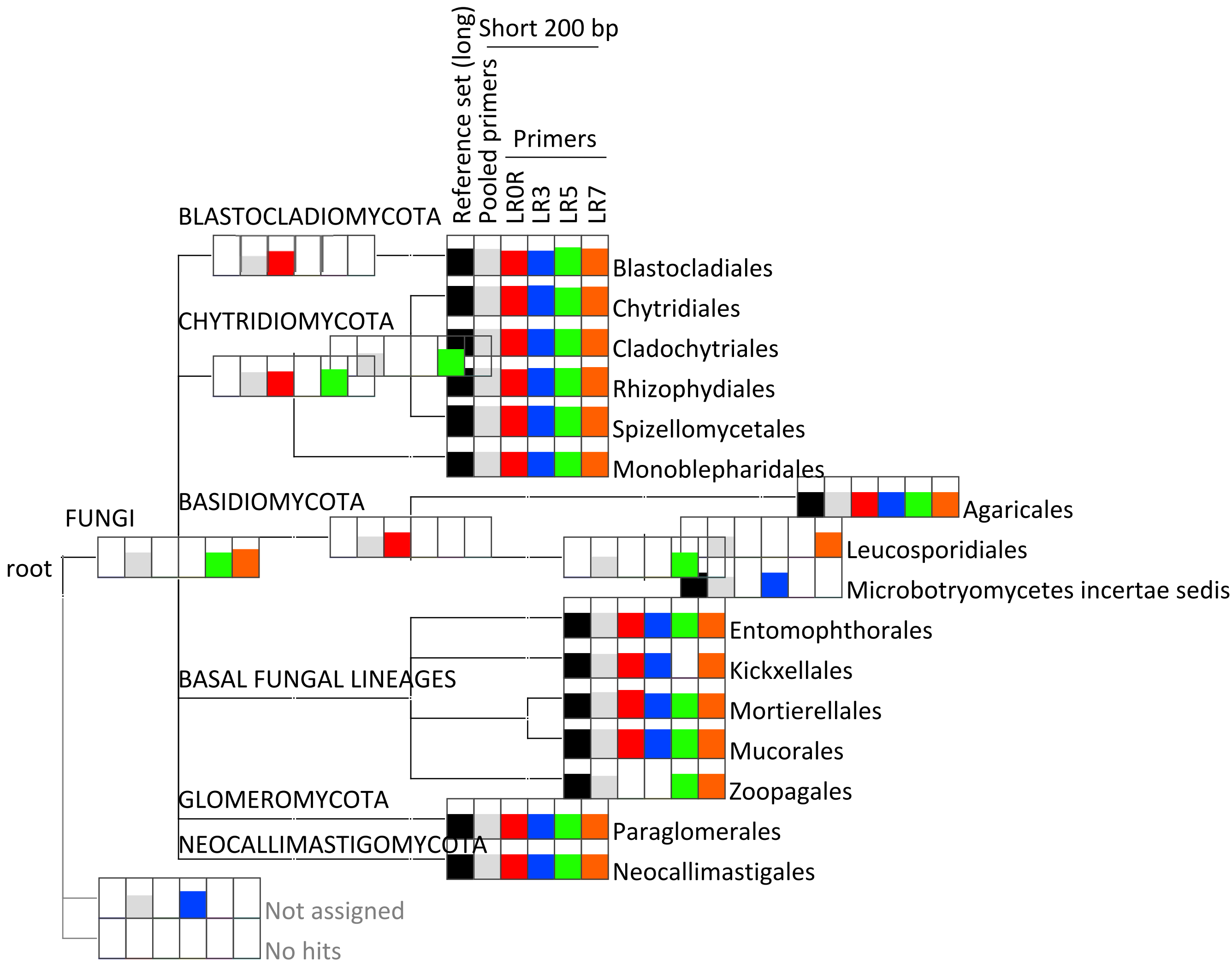

Supplement: Figure S4 — Taxonomic breakdown of non-metric multidimensional scaling community comparisons. Dataset sizes were normalized in MEGAN and taxonomic assignments of 200 bp sequences generated by four primers are compared with the reference set from Figure S1. In part (a), results are summarized at the species rank. In part (b), results are summarized at the order rank. In part (c), results are summarized at the order rank showing results for each primer and associated branch lengths using MEGAN. In each figure, boxes represent the relative number of sequences classified at each node/leaf and colors match those used in Figure 7 for each dataset (0% error). (PDF) [file pone.0035749.s004.pdf]

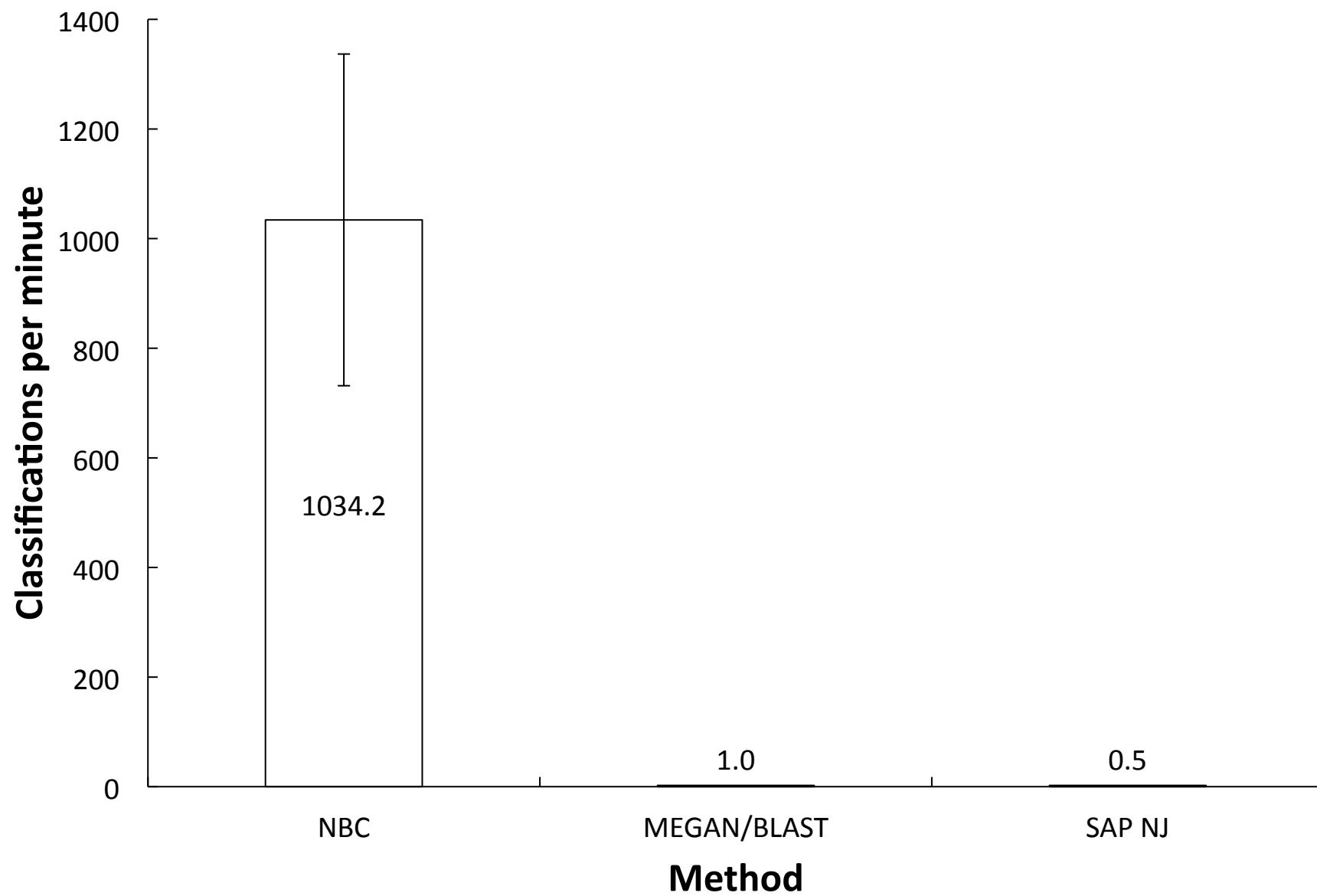

Supplement: Figure S5 — Number of classifications per minute. The average number of classifications per minute is shown for three methods. Bars indicate standard error of the mean using four different primers. For NBC, analysis times for all of our datasets was less than one minute. For BLAST + MEGAN, only the time to conduct local BLAST searches using a single processor was calculated, since MEGAN parsing with our data took less than a minute. With BLAST, the number of classifications per minute could be improved by using multiple processors for each search. For SAP, the total analysis time includes BLAST searches, homolog compilation, alignment, and neighbor joining analyses. (PDF) [file pone.0035749.s005.pdf]

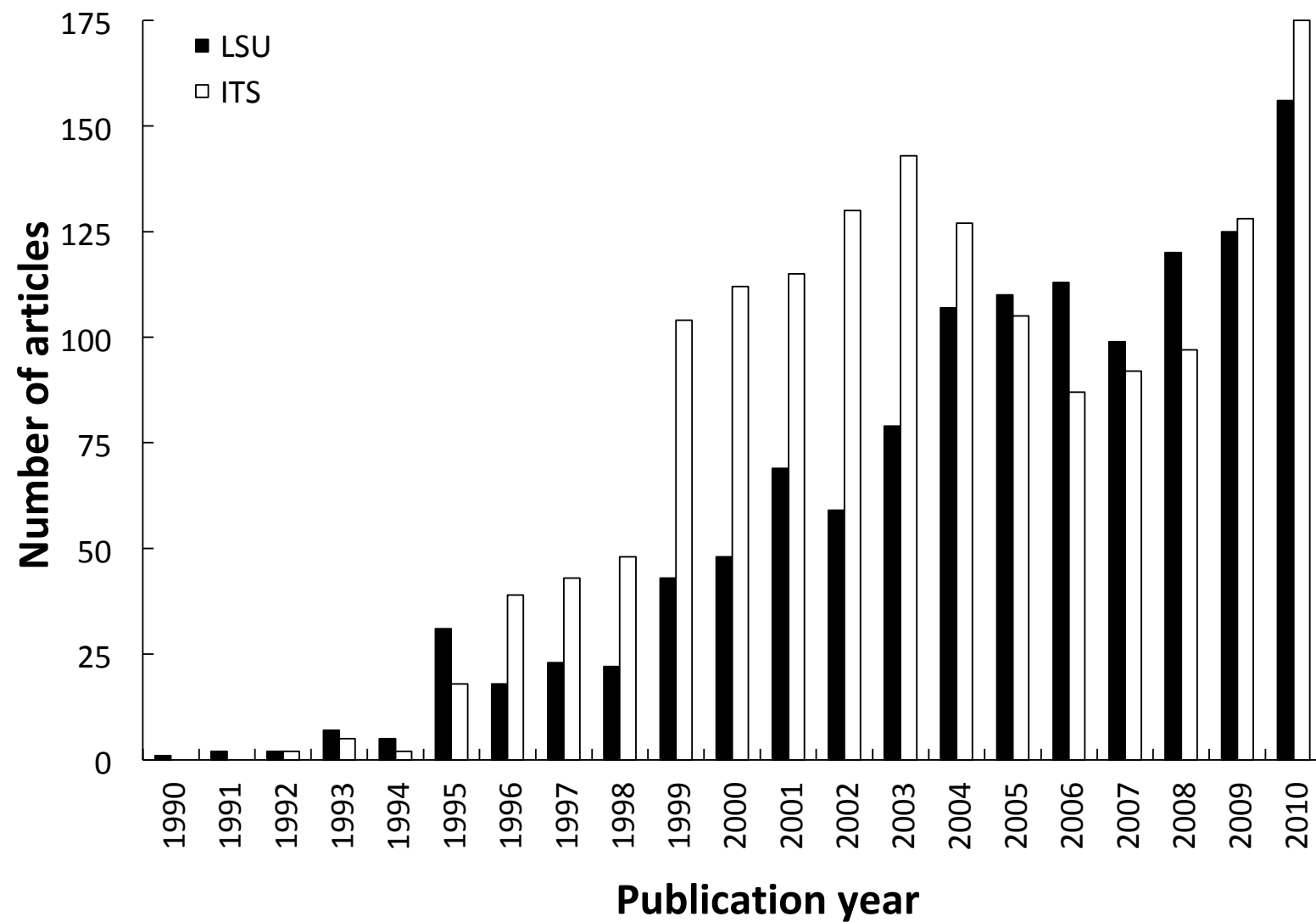

Supplement: Figure S6 — Articles indexed by Web of Knowledge from 1990–2010. Research articles with the topic of ITS (white) or LSU (black) phylogenetic systematics and/or barcoding are shown. (PDF) [file pone.0035749.s006.pdf]
