# Supplementary material for: Factors That Affect Large Subunit Ribosomal DNA Amplicon Sequencing Studies of Fungal Communities: Classification Method, Primer Choice, and Error
Source: PLoS One. 2012 Apr 27;7(4):e35749. doi: 10.1371/journal.pone.0035749 (PMC3338786; doi:10.1371/journal.pone.0035749)
Supplement: Table S1 — (DOC) [file pone.0035749.s007.doc]

Table S1: List of species and corresponding GenBank accessions included in the long LSU rDNA dataset.

| **Taxonomic Group** | **GenBank Accession** | **Species** | **Strain** | **Isolate** |
| --- | --- | --- | --- | --- |
| Ascomycota | | | | |
|  | AY584643 | Acarosporina microspora | CBS 338.39 | AFTOL-ID 78 |
|  | FJ176883 | Acremonium alternatum |  | AFTOL-ID 1396 |
|  | FJ176881 | Acremonium atrogriseum |  | AFTOL-ID 1394 |
|  | FJ176882 | Acremonium breve |  | AFTOL-ID 1395 |
|  | FJ176878 | Acremonium persicinum |  | AFTOL-ID 1391 |
|  | DQ986801 | Alectoria ochroleuca |  | AFTOL 209 |
|  | AY544654 | Aleuria aurantia | KH04012003-1 | AFTOL-ID 65 |
|  | DQ678082 | Alternaria alternata | CBS 916.96 | AFTOL-ID 1610 |
|  | DQ986756 | Amandinea punctata |  | AFTOL-ID 1306 |
|  | DQ470979 | Ambrosiella xylebori | CBS 110.61 | AFTOL-ID 1285 |
|  | FJ176889 | Amphilogia gyrosa |  | AFTOL-ID 1985 |
|  | FJ176863 | Amphisphaeria umbrina |  | AFTOL-ID 1229 |
|  | DQ883801 | Anaptychia palmatula |  | AFTOL-ID 648 |
|  | DQ782906 | Anisomeridium polypori |  | AFTOL-ID 101 |
|  | DQ836902 | Anthostomella torosa | JK 5678E | AFTOL-ID 732 |
|  | AY544660 | Anthracobia macrocystis | KH04042003-9 | AFTOL-ID 73 |
|  | DQ471018 | Apiospora montagnei | CBS 212.30 | AFTOL-ID 951 |
|  | FJ469668 | Arthonia caesia |  | AFTOL-ID 775 |
|  | FJ176864 | Arthrobotrys elegans |  | AFTOL-ID 1252 |
|  | EF413625 | Arthroderma ciferrii |  | AFTOL-ID 428 |
|  | DQ973029 | Asahinea scholanderi |  | AFTOL-ID 235 |
|  | AY544677 | Ascobolus carbonarius | KH-00-08 | AFTOL-ID 70 |
|  | AY544678 | Ascobolus crenulatus | KH.02.005 | AFTOL-ID 181 |
|  | FJ176886 | Ascocoryne sarcoides |  | AFTOL-ID 1834 |
|  | FJ176858 | Ascodesmis sphaerospora |  | AFTOL-ID 920 |
|  | FJ176897 | Aspergillus protuberus |  | AFTOL-ID 5007 |
|  | DQ986778 | Aspicilia caesiocinerea |  | AFTOL-ID 653 |
|  | DQ986779 | Aspicilia cinerea |  | AFTOL-ID 647 |
|  | DQ470956 | Aureobasidium pullulans | CBS 584.75 | AFTOL-ID 912 |
|  | DQ986793 | Bacidia rubella |  | AFTOL-ID 1793 |
|  | DQ782911 | Bacidia schweinitzii |  | AFTOL-ID 642 |
|  | DQ986798 | Bacidina arnoldiana |  | AFTOL-ID 1845 |
|  | EF643789 | Bagliettoa cazzae |  | AFTOL-ID 2227 |
|  | EF643805 | Bagliettoa parmigera |  | AFTOL-ID 2271 |
|  | EF643806 | Bagliettoa parmigerella |  | AFTOL-ID 2228 |
|  | EF643809 | Bagliettoa steineri |  | AFTOL-ID 2272 |
|  | DQ862027 | Bionectria ochroleuca |  | AFTOL-ID 187 |
|  | FJ176871 | Bisporella citrina |  | AFTOL-ID 1301 |
|  | DQ470970 | Bombardia bombarda |  | AFTOL-ID 967 |
|  | DQ986797 | Boreoplaca ultrafrigida |  | AFTOL-ID 1702 |
|  | DQ678051 | Botryosphaeria dothidea | CBS 115476 | AFTOL-ID 946 |
|  | DQ678064 | Botryosphaeria stevensii | CBS 431.82 | AFTOL-ID 1572 |
|  | DQ767655 | Botryosphaeria tsugae |  | AFTOL-ID 1586 |
|  | AY544651 | Botryotinia fuckeliana | spat 03-11 | AFTOL-ID 59 |
|  | DQ986751 | Bryoria trichodes |  | AFTOL-ID 205 |
|  | DQ973034 | Buellia fimbriata |  | AFTOL-ID 1051 |
|  | DQ883695 | Buellia frigida |  | AFTOL-ID 889 |
|  | DQ912342 | Buellia stillingiana |  | AFTOL-ID 571 |
|  | DQ470960 | Bulgaria inquinans | CBS 118.31 | AFTOL-ID 916 |
|  | DQ470987 | Caliciopsis orientalis | CBS 138.64 | AFTOL-ID 1911 |
|  | DQ678097 | Caliciopsis pinea | CBS 139.64 | AFTOL-ID 1869 |
|  | DQ247799 | Caloscypha fulgens | DJ053103-2 | AFTOL-ID 152 |
|  | DQ470941 | Camarops ustulinoides | DEH 2164 | AFTOL-ID 72 |
|  | DQ986791 | Candelaria concolor |  | AFTOL-ID 1706 |
|  | DQ912331 | Candelariella reflexa |  | AFTOL-ID 1271 |
|  | DQ986745 | Candelariella terrigena |  | AFTOL-ID 227 |
|  | AY584634 | Canoparmelia caroliniana |  | AFTOL-ID 6 |
|  | DQ247800 | Capnodium coffeae | CBS 147.52 | AFTOL-ID 939 |
|  | DQ678050 | Capnodium salicinum | CBS 131.34 | AFTOL-ID 937 |
|  | EF413604 | Capronia munkii |  | AFTOL-ID 656 |
|  | DQ823099 | Capronia pilosella |  | AFTOL-ID 657 |
|  | AY544685 | Carpoligna pleurothecii |  | AFTOL-ID 281 |
|  | EF643747 | Catapyrenium cinereum |  | AFTOL-ID 2230 |
|  | EF643748 | Catapyrenium daedaleum |  | AFTOL-ID 2273 |
|  | DQ678092 | Catenulostroma abietis | CBS 459.93 | AFTOL-ID 1789 |
|  | DQ986794 | Catolechia wahlenbergii |  | AFTOL-ID 1743 |
|  | EF413628 | Ceramothyrium carniolicum |  | AFTOL-ID 1063 |
|  | DQ678091 | Cercospora beticola | CBS 116456 | AFTOL-ID 1788 |
|  | DQ912334 | Cetraria islandica |  | AFTOL-ID 211 |
|  | AY544679 | Chaetomella acutiseta | DAOM 230096 | AFTOL-ID 270 |
|  | AY544661 | Cheilymenia stercorea | KH04282003-4 | AFTOL-ID 148 |
|  | DQ986782 | Circinaria contorta |  | AFTOL-ID 1358 |
|  | AY584640 | Cladonia caroliniana |  | AFTOL-ID 3 |
|  | DQ973026 | Cladonia stipitata |  | AFTOL-ID 1657 |
|  | DQ678057 | Cladosporium cladosporioides | CBS 170.54 | AFTOL-ID 1289 |
|  | EF643749 | Clavascidium umbrinum |  | AFTOL-ID 2274 |
|  | FJ176872 | Clavispora lusitaniae |  | AFTOL-ID 1318 |
|  | DQ912346 | Coccocarpia domingensis |  | AFTOL-ID 122 |
|  | DQ883800 | Coccocarpia erythroxyli |  | AFTOL-ID 333 |
|  | AY544657 | Coccomyces dentatus | KH04042003-2 | AFTOL-ID 147 |
|  | DQ470975 | Coccomyces strobi | CBS 202.91 | AFTOL-ID 1250 |
|  | AY544645 | Cochliobolus heterostrophus | CBS 134.39 | AFTOL-ID 54 |
|  | DQ678045 | Cochliobolus sativus | DAOM 226212 | AFTOL-ID 271 |
|  | DQ917408 | Collema cristatum |  | AFTOL-ID 1013 |
|  | DQ470959 | Coniochaeta ostrea | CBS 507.70 | AFTOL-ID 915 |
|  | DQ678054 | Coniothyrium obiones | CBS 453.68 | AFTOL-ID 1240 |
|  | DQ767653 | Coniothyrium palmarum |  | AFTOL-ID 1379 |
|  | FJ176866 | Connersia rilstonii |  | AFTOL-ID 1264 |
|  | FJ176900 | Corollospora angusta |  | AFTOL-ID 5010 |
|  | FJ176901 | Corollospora maritima |  | AFTOL-ID 5011 |
|  | AY544680 | Crinula caliciiformis | DAOM 231117 | AFTOL-ID 272 |
|  | AY584653 | Crocynia pyxinoides |  | AFTOL-ID 111 |
|  | DQ862028 | Cryptosporella hypodermia |  | AFTOL-ID 2124 |
|  | DQ678061 | Cucurbitaria elongata | CBS 171.55 | AFTOL-ID 1568 |
|  | DQ470944 | Cudoniella clavus |  | AFTOL-ID 166 |
|  | EF413619 | Cyphellophora laciniata |  | AFTOL-ID 1033 |
|  | DQ986802 | Dactylina arctica |  | AFTOL 225 |
|  | FJ176855 | Dactylospora haliotrepha |  | AFTOL-ID 758 |
|  | FJ713617 | Dactylospora haliotrepha | JK 5129B | AFTOL-ID 798 |
|  | FJ176896 | Dactylospora imperfecta |  | AFTOL-ID 5006 |
|  | FJ176891 | Dactylospora lobariella |  | AFTOL-ID 2137 |
|  | FJ176890 | Dactylospora mangrovei |  | AFTOL-ID 2108 |
|  | DQ678074 | Davidiella tassiana | CBS 399.80 | AFTOL-ID 1591 |
|  | DQ912347 | Degelia plumbea |  | AFTOL-ID 990 |
|  | DQ678077 | Delitschia winteri | CBS 225.62 | AFTOL-ID 1599 |
|  | DQ470977 | Delphinella strobiligena | CBS 735.71 | AFTOL-ID 1257 |
|  | DQ470971 | Dendryphiella arenaria | CBS 181.58 | AFTOL-ID 995 |
|  | DQ678046 | Dendryphiopsis atra | DAOM 231155 | AFTOL-ID 273 |
|  | EF643750 | Dermatocarpon luridum |  | AFTOL-ID 2277 |
|  | AY584644 | Dermatocarpon miniatum |  | AFTOL-ID 91 |
|  | DQ247801 | Dermea acerina | CBS 161.38 | AFTOL-ID 941 |
|  | DQ470964 | Diatrype disciformis | CBS 197.49 | AFTOL-ID 927 |
|  | EF177845 | Didymella exigua |  | AFTOL-ID 2111 |
|  | DQ678070 | Didymella pisi | CBS 126.54 | AFTOL-ID 1583 |
|  | DQ678079 | Didymosphaeria enalia | CBS 304.66 | AFTOL-ID 1601 |
|  | DQ883799 | Diploschistes cinereocaesius |  | AFTOL-ID 328 |
|  | DQ973035 | Dirinaria applanata |  | AFTOL-ID 839 |
|  | AY544667 | Disciotis venosa | NRRL 22213 | AFTOL-ID 179 |
|  | DQ836907 | Doratomyces stemonitis | CBS 127.22 | AFTOL-ID 1380 |
|  | DQ678048 | Dothidea hippophaeos | CBS 188.58 | AFTOL-ID 919 |
|  | DQ247802 | Dothidea insculpta | CBS 189.58 | AFTOL-ID 921 |
|  | AY544681 | Dothidea sambuci | DAOM 231303 | AFTOL-ID 274 |
|  | DQ470984 | Dothiora cannabinae | CBS 737.71 | AFTOL-ID 1359 |
|  | DQ782905 | Echinoplaca strigulacea |  | AFTOL-ID 106 |
|  | DQ470966 | Eleutherascus lectardii | CBS 626.71 | AFTOL-ID 933 |
|  | DQ678094 | Elsinoe centrolobi | CBS 222.50 | AFTOL-ID 1854 |
|  | DQ678095 | Elsinoe phaseoli | CBS 165.31 | AFTOL-ID 1855 |
|  | DQ767658 | Elsinoe veneta |  | AFTOL-ID 1853 |
|  | DQ678060 | Elsinoe veneta | CBS 164.29 | AFTOL-ID 1360 |
|  | FJ176861 | Emericellopsis maritima |  | AFTOL-ID 999 |
|  | EF643751 | Endocarpon adscendens |  | AFTOL-ID 2231 |
|  | EF643773 | Endocarpon diffractellum |  | AFTOL-ID 2241 |
|  | DQ823097 | Endocarpon pallidulum |  | AFTOL-ID 661 |
|  | EF643752 | Endocarpon petrolepideum |  | AFTOL-ID 2232 |
|  | EF643753 | Endocarpon psorodeum |  | AFTOL-ID 2233 |
|  | EF643754 | Endocarpon pusillum |  | AFTOL-ID 2279 |
|  | DQ470972 | Endothia gyrosa | CBS 112915 | AFTOL-ID 1223 |
|  | DQ973041 | Erioderma verruculosum |  | AFTOL-ID 337 |
|  | EF413621 | Eupenicillium javanicum |  | AFTOL-ID 429 |
|  | EF411064 | Eupenicillium limosum |  | AFTOL-ID 2014 |
|  | DQ836903 | Eutypa lata | CBS 208.87 | AFTOL-ID 929 |
|  | DQ823100 | Exophiala dermatitidis |  | AFTOL-ID 668 |
|  | DQ823101 | Exophiala pisciphila |  | AFTOL-ID 669 |
|  | EF413609 | Exophiala salmonis |  | AFTOL-ID 671 |
|  | DQ973045 | Fissurina insidiosa |  | AFTOL-ID 1662 |
|  | DQ883795 | Flavocetraria nivalis |  | AFTOL-ID 231 |
|  | AY584639 | Flavoparmelia caperata |  | AFTOL-ID 2 |
|  | DQ912335 | Flavopunctelia flaventior |  | AFTOL-ID 317 |
|  | DQ917417 | Fuscopannaria ignobilis |  | AFTOL-ID 1011 |
|  | DQ917419 | Fuscopannaria mediterranea |  | AFTOL-ID 1014 |
|  | FJ176854 | Gaeumannomyces medullaris |  | AFTOL-ID 734 |
|  | FJ176869 | Galiella rufa |  | AFTOL-ID 1297 |
|  | DQ470980 | Gelasinospora tetrasperma | CBS 178.33 | AFTOL-ID 1287 |
|  | AY544650 | Geoglossum nigritum | spat 03-08 | AFTOL-ID 56 |
|  | FJ176880 | Gliomastix murorum |  | AFTOL-ID 1393 |
|  | DQ247803 | Glomerobolus gelineus | JK 5548K | AFTOL-ID 1349 |
|  | FJ176888 | Gondwanamyces capensis |  | AFTOL-ID 1907 |
|  | DQ836906 | Graphostroma platystoma | CBS 270.87 | AFTOL-ID 1249 |
|  | DQ678085 | Guignardia bidwellii | CBS 237.48 | AFTOL-ID 1618 |
|  | DQ678089 | Guignardia gaultheriae | CBS 447.70 | AFTOL-ID 1784 |
|  | DQ973046 | Gyalidea hyalinus |  | AFTOL-ID 332 |
|  | AY584649 | Gyalideopsis vulgaris |  | AFTOL-ID 105 |
|  | AY544673 | Gyromitra californica | 06/22/2003 | AFTOL-ID 176 |
|  | FJ176906 | Gyromitra esculenta |  | AFTOL-ID 5017 |
|  | DQ678083 | Helicomyces roseus | CBS 283.51 | AFTOL-ID 1613 |
|  | DQ678071 | Herpotrichia diffusa | CBS 250.62 | AFTOL-ID 1588 |
|  | DQ678080 | Herpotrichia juniperi | CBS 200.31 | AFTOL-ID 1608 |
|  | DQ883798 | Heterodermia vulgaris |  | AFTOL-ID 320 |
|  | EF643755 | Heteroplacidium contumescens |  | AFTOL-ID 2280 |
|  | EF643756 | Heteroplacidium imbricatum |  | AFTOL-ID 2281 |
|  | EF643783 | Hydropunctaria adriatica |  | AFTOL-ID 2251 |
|  | EF643801 | Hydropunctaria maura |  | AFTOL-ID 2263 |
|  | EF643808 | Hydropunctaria rheitrophila |  | AFTOL-ID 2266 |
|  | DQ782914 | Hypocenomyce scalaris |  | AFTOL-ID 687 |
|  | AY544649 | Hypocrea americana | spat 03-04 | AFTOL-ID 52 |
|  | DQ973030 | Hypogymnia physodes |  | AFTOL-ID 195 |
|  | DQ912336 | Hypotrachyna caraccensis |  | AFTOL-ID 312 |
|  | DQ912337 | Hypotrachyna degelii |  | AFTOL-ID 324 |
|  | DQ678055 | Hysterium pulicare | CBS 239.34 | AFTOL-ID 1254 |
|  | DQ767657 | Hysteropatella elliptica |  | AFTOL-ID 1790 |
|  | DQ883694 | Icmadophila ericetorum |  | AFTOL-ID 875 |
|  | DQ986753 | Imshaugia aleurites |  | AFTOL-ID 1044 |
|  | EF177846 | Julella avicenniae |  | AFTOL-ID 2205 |
|  | FJ176893 | Knufia chersonesos |  | AFTOL-ID 2195 |
|  | AY544646 | Lachnum virgineum | spat 03-01 | AFTOL-ID 49 |
|  | DQ470978 | Lambertella subrenispora | CBS 811.85 | AFTOL-ID 1262 |
|  | DQ883691 | Lasallia papulosa |  | AFTOL-ID 650 |
|  | DQ883690 | Lasallia pustulata |  | AFTOL-ID 554 |
|  | FJ176873 | Lasiobolidium spirale |  | AFTOL-ID 1321 |
|  | DQ973027 | Lecanora achariana |  | AFTOL-ID 1693 |
|  | DQ986746 | Lecanora contractula |  | AFTOL-ID 877 |
|  | DQ782910 | Lecanora hybocarpa |  | AFTOL-ID 639 |
|  | DQ986792 | Lecanora polytropa |  | AFTOL-ID 1798 |
|  | DQ912332 | Lecidea fuscoatra |  | AFTOL-ID 589 |
|  | DQ986747 | Lecidella elaeochroma |  | AFTOL-ID 1275 |
|  | DQ986759 | Lecidoma demissum |  | AFTOL-ID 1376 |
|  | AY544644 | Leotia lubrica | spat 03-012 | AFTOL-ID 1 |
|  | DQ678067 | Lepidosphaeria nicotiae | CBS 101341 | AFTOL-ID 1576 |
|  | DQ986795 | Lepraria incana |  | AFTOL-ID 1792 |
|  | DQ986768 | Lepraria lobificans |  | AFTOL-ID 325 |
|  | DQ917412 | Leptogium lichenoides |  | AFTOL-ID 1015 |
|  | DQ470946 | Leptosphaeria maculans | DAOM 229267 | AFTOL-ID 277 |
|  | AY584648 | Letrouitia domingensis |  | AFTOL-ID 102 |
|  | FJ176884 | Leuconeurospora pulcherrima |  | AFTOL-ID 1397 |
|  | DQ678044 | Lewia eureka | DAOM 195275 | AFTOL-ID 267 |
|  | DQ782916 | Lichinella iodopulchra |  | AFTOL-ID 896 |
|  | FJ176902 | Lindra obtusa |  | AFTOL-ID 5012 |
|  | DQ470947 | Lindra thalassiae | JK 5090A | AFTOL-ID 413 |
|  | FJ176875 | Lipomyces oligophaga |  | AFTOL-ID 1323 |
|  | AY584638 | Lithothelium septemseptatum |  | AFTOL-ID 12 |
|  | AY584655 | Lobaria scrobiculata |  | AFTOL-ID 128 |
|  | DQ883796 | Lobariella pallida |  | AFTOL-ID 310 |
|  | AY584651 | Lopezaria versicolor |  | AFTOL-ID 108 |
|  | DQ782384 | Lophiostoma arundinis |  | AFTOL-ID 1606 |
|  | DQ678069 | Lophiostoma crenatum | CBS 629.86 | AFTOL-ID 1581 |
|  | DQ678081 | Lophium mytilinum | CBS 269.34 | AFTOL-ID 1609 |
|  | DQ470957 | Loramyces macrosporus | CBS 235.53 | AFTOL-ID 913 |
|  | DQ986749 | Loxospora cismonica |  | AFTOL-ID 878 |
|  | DQ986750 | Loxospora ochrophaea |  | AFTOL-ID 879 |
|  | FJ176904 | Lulwoana uniseptata |  | AFTOL-ID 5014 |
|  | DQ522856 | Lulworthia grandispora |  | AFTOL-ID 424 |
|  | FJ176903 | Lulworthia lignoarenaria |  | AFTOL-ID 5013 |
|  | DQ678088 | Macrophomina phaseolina | CBS 227.33 | AFTOL-ID 1783 |
|  | DQ470943 | Marcelleina persoonii |  | AFTOL-ID 164 |
|  | DQ973031 | Masonhalea richardsonii |  | AFTOL-ID 1710 |
|  | AY584650 | Megalospora tuberculosa |  | AFTOL-ID 107 |
|  | FJ713618 | Melanconis stilbostoma |  | AFTOL-ID 936 |
|  | DQ986803 | Melanelixia fuliginosa |  | AFTOL 1370 |
|  | AY584637 | Menegazzia terebrata |  | AFTOL-ID 10 |
|  | AY544682 | Menispora tortuosa | DAOM 231154 | AFTOL-ID 278 |
|  | DQ470954 | Meria laricis | CBS 298.52 | AFTOL-ID 907 |
|  | FJ176876 | Metschnikowia bicuspidata |  | AFTOL-ID 1326 |
|  | DQ470958 | Microascus trigonosporus | CBS 218.31 | AFTOL-ID 914 |
|  | DQ470981 | Microglossum rufum |  | AFTOL-ID 1292 |
|  | DQ470942 | Mollisia cinerea |  | AFTOL-ID 76 |
|  | DQ782908 | Monascus purpureus |  | AFTOL-ID 426 |
|  | AY544683 | Monilinia fructicola |  | AFTOL-ID 279 |
|  | AY544670 | Monilinia laxa |  | AFTOL-ID 169 |
|  | DQ678086 | Montagnula opulenta | CBS 168.34 | AFTOL-ID 1734 |
|  | DQ782915 | Mycoblastus sanguinarius |  | AFTOL-ID 196 |
|  | DQ678098 | Mycosphaerella fijiensis | OSC 100622 | AFTOL-ID 2021 |
|  | DQ678084 | Mycosphaerella graminicola | CBS 292.38 | AFTOL-ID 1615 |
|  | FJ176856 | Mycosphaerella pneumatophorae |  | AFTOL-ID 762 |
|  | DQ470968 | Mycosphaerella punctiformis | CBS 113265 | AFTOL-ID 942 |
|  | DQ973025 | Myelochroa aurulenta |  | AFTOL-ID 206 |
|  | DQ678059 | Myriangium duriaei | CBS 260.36 | AFTOL-ID 1304 |
|  | FJ176867 | Naemacyclus fimbriatus |  | AFTOL-ID 1295 |
|  | FJ176868 | Naemacyclus minor |  | AFTOL-ID 1296 |
|  | FJ176865 | Neobulgaria pura |  | AFTOL-ID 1259 |
|  | EF643757 | Neocatapyrenium rhizinosum |  | AFTOL-ID 2282 |
|  | AY544662 | Neofabraea malicorticis | CLS003 | AFTOL-ID 149 |
|  | DQ678053 | Neofusicoccum ribis | CBS 115475 | AFTOL-ID 1232 |
|  | DQ470986 | Neolecta irregularis |  | AFTOL-ID 1363 |
|  | DQ470985 | Neolecta vitellina |  | AFTOL-ID 1362 |
|  | DQ973040 | Nephroma arcticum |  | AFTOL-ID 1711 |
|  | AY584656 | Nephroma parile |  | AFTOL-ID 131 |
|  | DQ986762 | Niebla cephalota |  | AFTOL-ID 777 |
|  | FJ176892 | Nigrospora oryzae |  | AFTOL-ID 2179 |
|  | DQ986776 | Ochrolechia yasudae |  | AFTOL-ID 882 |
|  | DQ973028 | Ophioparma lapponica |  | AFTOL-ID 1707 |
|  | DQ767656 | Ophiosphaerella herpotricha |  | AFTOL-ID 1595 |
|  | DQ678062 | Ophiosphaerella herpotricha | CBS 620.86 | AFTOL-ID 1569 |
|  | DQ470955 | Ophiostoma piliferum | CBS 158.74 | AFTOL-ID 910 |
|  | DQ836904 | Ophiostoma stenoceras | CBS 139.51 | AFTOL-ID 1038 |
|  | DQ470953 | Orbilia auricolor | CBS 547.63 | AFTOL-ID 906 |
|  | DQ470952 | Orbilia vinosa | CBS 917.72 | AFTOL-ID 905 |
|  | AY584642 | Ostropa barbara | CBS 260.58 | AFTOL-ID 77 |
|  | FJ176852 | Pachyella babingtonii |  | AFTOL-ID 57 |
|  | DQ470950 | Papulosa amerospora | JK 5547F | AFTOL-ID 748 |
|  | EF643792 | Parabagliettoa dufourii |  | AFTOL-ID 2254 |
|  | DQ912338 | Parmotrema austrosinense |  | AFTOL-ID 89 |
|  | DQ912339 | Parmotrema reticulatum |  | AFTOL-ID 8 |
|  | AY584635 | Parmotrema tinctorum |  | AFTOL-ID 7 |
|  | AY584657 | Peltigera degenii |  | AFTOL-ID 134 |
|  | DQ782907 | Pertusaria dactylina |  | AFTOL-ID 224 |
|  | DQ470969 | Petriella setifera | CBS 437.75 | AFTOL-ID 956 |
|  | DQ470967 | Pezicula carpinea | CBS 282.39 | AFTOL-ID 938 |
|  | AY544659 | Peziza proteana f. sparassoides | KH04042003-7 | AFTOL-ID 71 |
|  | DQ470948 | Peziza vesiculosa |  | AFTOL-ID 507 |
|  | DQ470976 | Phacidium lacerum | CBS 130.30 | AFTOL-ID 1253 |
|  | DQ678073 | Phaeodothis winteri | CBS 182.58 | AFTOL-ID 1590 |
|  | DQ912343 | Phaeophyscia orbicularis |  | AFTOL-ID 1308 |
|  | AY544684 | Phaeosphaeria avenaria |  | AFTOL-ID 280 |
|  | DQ678063 | Phaeosphaeria eustoma | CBS 573.86 | AFTOL-ID 1570 |
|  | EF177847 | Phaeosphaeria olivacea |  | AFTOL-ID 2206 |
|  | EF179158 | Phaeosphaeria orae-maris |  | AFTOL-ID 1441 |
|  | EF413615 | Phialophora verrucosa |  | AFTOL-ID 670 |
|  | DQ986771 | Phlyctis argena |  | AFTOL-ID 1375 |
|  | DQ678066 | Phoma herbarum | CBS herbarum | AFTOL-ID 1575 |
|  | FJ176857 | Phomatospora bellaminuta |  | AFTOL-ID 766 |
|  | DQ986780 | Phyllobaeis erythrella |  | AFTOL-ID 329 |
|  | DQ986781 | Phyllobaeis imbricata |  | AFTOL-ID 852 |
|  | DQ678090 | Phyllosticta flevolandica | CBS 998.72 | AFTOL-ID 1786 |
|  | DQ782904 | Physcia aipolia |  | AFTOL-ID 84 |
|  | DQ912344 | Physconia muscigena |  | AFTOL-ID 220 |
|  | EF643758 | Placidiopsis cartilaginea |  | AFTOL-ID 2283 |
|  | EF643759 | Placidiopsis cinerascens |  | AFTOL-ID 2284 |
|  | EF643760 | Placidium acarosporoides |  | AFTOL-ID 2234 |
|  | EF643765 | Placidium arboreum |  | AFTOL-ID 2285 |
|  | EF643761 | Placidium lachneum |  | AFTOL-ID 2286 |
|  | EF643762 | Placidium lacinulatum |  | AFTOL-ID 2236 |
|  | EF643764 | Placidium squamulosum |  | AFTOL-ID 2288 |
|  | EF643766 | Placocarpus schaereri |  | AFTOl-ID 2289 |
|  | EF643768 | Placopyrenium bucekii |  | AFTOL-ID 2238 |
|  | EF643784 | Placopyrenium canellum |  | AFTOL-ID 2252 |
|  | EF643794 | Placopyrenium fuscellum |  | AFTOL-ID 2256 |
|  | DQ986774 | Placynthiella uliginosa |  | AFTOL-ID 1365 |
|  | DQ973047 | Placynthium flabellosum |  | AFTOL-ID 1663 |
|  | DQ836905 | Plagiostoma aesculi | CBS 109765 | AFTOL-ID 1238 |
|  | DQ912340 | Platismatia glauca |  | AFTOL-ID 203 |
|  | DQ678078 | Pleomassaria siparia | CBS 279.74 | AFTOL-ID 1600 |
|  | DQ842017 | Pleopsidium chlorophanum |  | AFTOL-ID 1004 |
|  | DQ883698 | Pleopsidium gobiense |  | AFTOL-ID 1003 |
|  | DQ247804 | Pleospora herbarum | CBS 191.86 | AFTOL-ID 940 |
|  | DQ678049 | Pleospora herbarum | CBS 714.68 | AFTOL-ID 934 |
|  | DQ842029 | Plicaria leiocarpa |  | AFTOL-ID 1345 |
|  | EF643769 | Polyblastia cupularis |  | AFTOL-ID 2239 |
|  | EF413601 | Polyblastia melaspora |  | AFTOL-ID 1356 |
|  | EF643771 | Polyblastia viridescens |  | AFTOL-ID 2240 |
|  | DQ986770 | Polychidium muscicola |  | AFTOL-ID 230 |
|  | DQ986757 | Porpidia albocaerulescens |  | AFTOL-ID 1246 |
|  | DQ986758 | Porpidia speirea |  | AFTOL-ID 1050 |
|  | DQ470949 | Potebniamyces pyri | S 001 | AFTOL-ID 744 |
|  | DQ678056 | Preussia minima | CBS 524.50 | AFTOL-ID 1256 |
|  | AY544686 | Preussia terricola |  | AFTOL-ID 282 |
|  | DQ912350 | Protopannaria pezizoides |  | AFTOL-ID 222 |
|  | DQ470988 | Pseudeurotium zonatum | CBS 329.36 | AFTOL-ID 1912 |
|  | DQ986754 | Pseudevernia consocians |  | AFTOL-ID 1243 |
|  | DQ883794 | Pseudocyphellaria anomala |  | AFTOL-ID 132 |
|  | AY544658 | Pseudopithyella minuscula | KH04042003-1 | AFTOL-ID 69 |
|  | DQ986760 | Psora decipiens |  | AFTOL-ID 1032 |
|  | DQ986761 | Psora rubiformis |  | AFTOL-ID 219 |
|  | AY584646 | Punctelia hypoleucites |  | AFTOL-ID 85 |
|  | AY584636 | Punctelia rudecta |  | AFTOL-ID 9 |
|  | DQ986800 | Pycnothelia papillaria |  | AFTOL 1377 |
|  | DQ678096 | Pyrenochaeta nobilis | CBS 407.76 | AFTOL-ID 1856 |
|  | DQ499596 | Pyrenophora phaeocomes |  | AFTOL-ID 283 |
|  | AY544672 | Pyrenophora tritici-repentis |  | AFTOL-ID 173 |
|  | EF411063 | Pyrenula aspistea |  | AFTOL-ID 2012 |
|  | DQ823103 | Pyrgillus javanicus |  | AFTOL-ID 342 |
|  | DQ247805 | Pyronema domesticum | CBS 666.88 | AFTOL-ID 949 |
|  | FJ176894 | Pyxidiophora arvernensis |  | AFTOL-ID 2197 |
|  | DQ973036 | Pyxine sorediata |  | AFTOL-ID 207 |
|  | DQ883802 | Pyxine subcinerea |  | AFTOL-ID 686 |
|  | DQ883783 | Ramalina complanata |  | AFTOL-ID 966 |
|  | DQ823102 | Ramichloridium anceps |  | AFTOL-ID 659 |
|  | DQ470961 | Rhizina undulata | CBS 300.56 | AFTOL-ID 918 |
|  | DQ986804 | Rhizocarpon oederi |  | AFTOL 1372 |
|  | FJ469672 | Rhytidhysteron rufulum | CBS 306.38 | AFTOL-ID 2109 |
|  | DQ912345 | Rinodina tephraspis |  | AFTOL-ID 1314 |
|  | AY584654 | Roccella fuciformis |  | AFTOL-ID 126 |
|  | DQ883696 | Roccellographa cretacea |  | AFTOL-ID 93 |
|  | DQ470963 | Rutstroemia firma | CBS 341.62 | AFTOL-ID 923 |
|  | FJ176870 | Saccobolus dilutellus |  | AFTOL-ID 1299 |
|  | AY544647 | Sarcoscypha coccinea | spat 03-02 | AFTOL-ID 50 |
|  | FJ176859 | Sarcoscypha coccinea |  | AFTOL-ID 930 |
|  | FJ176860 | Sarcosoma latahense |  | AFTOL-ID 954 |
|  | AY544668 | Sarcosphaera crassa | KH05242003-4 | AFTOL-ID 153 |
|  | FJ176879 | Sarocladium strictum |  | AFTOL-ID 1392 |
|  | DQ470965 | Sclerotinia sclerotiorum | CBS 499.50 | AFTOL-ID 928 |
|  | DQ678075 | Scorias spongiosa | CBS 325.33 | AFTOL-ID 1594 |
|  | DQ247806 | Scutellinia scutellata | KH03212003-1 | AFTOL-ID 62 |
|  | FJ176898 | Sigmoidea marina |  | AFTOL-ID 5008 |
|  | DQ986775 | Siphula ceratites |  | AFTOL-ID 849 |
|  | DQ973043 | Solorina crocea |  | AFTOL-ID 1619 |
|  | DQ973044 | Solorina saccata |  | AFTOL-ID 127 |
|  | FJ997861 | Spathularia velutipes |  | AFTOL-ID 1291 |
|  | DQ678087 | Spencermartinsia viticola | CBS 117009 | AFTOL-ID 1782 |
|  | DQ986805 | Sphaerophorus fragilis |  | AFTOL 226 |
|  | DQ986767 | Sphaerophorus globosus |  | AFTOL-ID 1057 |
|  | EF413632 | Sphinctrina turbinata |  | AFTOL-ID 1721 |
|  | DQ782909 | Spiromastix warcupii |  | AFTOL-ID 430 |
|  | DQ678065 | Splanchnonema platani | CBS 221.37 | AFTOL-ID 1574 |
|  | DQ986763 | Squamarina cartilaginea |  | AFTOL-ID 1281 |
|  | EF643772 | Staurothele areolata |  | AFTOL-ID 2291 |
|  | EF643774 | Staurothele drummondii |  | AFTOL-ID 2242 |
|  | EF643775 | Staurothele fissa |  | AFTOL-ID 2243 |
|  | DQ823098 | Staurothele frustulenta |  | AFTOL-ID 697 |
|  | EF643776 | Staurothele immersa |  | AFTOL-ID 2244 |
|  | DQ986769 | Sticta beauvoisii |  | AFTOL-ID 1242 |
|  | AY544675 | Sydowia polyspora | CLS-10 | AFTOL-ID 178 |
|  | DQ678058 | Sydowia polyspora | CBS 16.29 | AFTOL-ID 1300 |
|  | FJ176887 | Symbiotaphrina buchneri |  | AFTOL-ID 1836 |
|  | DQ470973 | Taphrina deformans | CBS 356.35 | AFTOL-ID 1234 |
|  | AY584647 | Teloschistes exilis |  | AFTOL-ID 87 |
|  | DQ986764 | Tephromela atra |  | AFTOL-ID 780 |
|  | FJ176895 | Thelebolus ellipsoideus | dH11718 | AFTOL-ID 5005 |
|  | FJ176905 | Thelebolus globosus |  | AFTOL-ID 5016 |
|  | EF643778 | Thelidium decipiens |  | AFTOL-ID 2246 |
|  | EF643780 | Thelidium incavatum |  | AFTOL-ID 2248 |
|  | EF643781 | Thelidium papulare |  | AFTOL-ID 2249 |
|  | EF643782 | Thelidium pyrenophorum |  | AFTOL-ID 2250 |
|  | AY544671 | Thyridium vestitum | AR3872 | AFTOL-ID 172 |
|  | DQ973039 | Toninia sedifolia |  | AFTOL-ID 213 |
|  | DQ973037 | Tornabea scutellifera |  | AFTOL-ID 1061 |
|  | DQ470951 | Torpedospora radiata | JK 5095A | AFTOL-ID 751 |
|  | DQ678072 | Trematosphaeria pertusa | CBS 400.97 | AFTOL-ID 1589 |
|  | AY544653 | Trichoglossum hirsutum | KH03232003-1 | AFTOL-ID 64 |
|  | DQ470983 | Tryblidiopsis pinastri | CBS 445.71 | AFTOL-ID 1319 |
|  | FJ176877 | Tuber gibbosum |  | AFTOL-ID 1344 |
|  | DQ470982 | Tubeufia cerea | CBS 254.75 | AFTOL-ID 1316 |
|  | DQ767654 | Tubeufia helicomyces |  | AFTOL-ID 1580 |
|  | DQ986755 | Tuckermannopsis ciliaris |  | AFTOL-ID 1245 |
|  | DQ470974 | Tyrannosorus pinicola | CBS 124.88 | AFTOL-ID 1235 |
|  | DQ678076 | Ulospora bilgramii | CBS 101364 | AFTOL-ID 1598 |
|  | DQ986799 | Umbilicaria aprina |  | AFTOL 1416 |
|  | DQ986772 | Umbilicaria arctica |  | AFTOL-ID 1266 |
|  | DQ782912 | Umbilicaria mammulata |  | AFTOL-ID 645 |
|  | DQ986773 | Umbilicaria spodochroa |  | AFTOL-ID 555 |
|  | DQ883692 | Usnea antarctica |  | AFTOL-ID 813 |
|  | DQ883693 | Usnea sphacelata |  | AFTOL-ID 816 |
|  | DQ973033 | Usnea strigosa |  | AFTOL-ID 5 |
|  | FJ176853 | Verpa bohemica |  | AFTOL-ID 58 |
|  | AY544666 | Verpa conica | NRRL 22338 | AFTOL-ID 74 |
|  | EF643786 | Verrucaria baldensis |  | AFTOL-ID 2270 |
|  | EF643787 | Verrucaria caerulea |  | AFTOL-ID 2293 |
|  | EF643790 | Verrucaria cyanea |  | AFTOL-ID 2294 |
|  | EF643791 | Verrucaria dolosa |  | AFTOL-ID 2253 |
|  | EF643793 | Verrucaria fuscula |  | AFTOL-ID 2255 |
|  | EF643795 | Verrucaria hochstetteri |  | AFTOL-ID 2257 |
|  | EF643796 | Verrucaria hydrela |  | AFTOL-ID 2259 |
|  | EF643797 | Verrucaria latebrosa |  | AFTOL-ID 2260 |
|  | EF643798 | Verrucaria lecideoides |  | AFTOL-ID 2295 |
|  | EF643799 | Verrucaria macrostoma |  | AFTOL-ID 2261 |
|  | EF643800 | Verrucaria marmorea |  | AFTOL-ID 2262 |
|  | EF643803 | Verrucaria muralis |  | AFTOL-ID 2265 |
|  | EF643804 | Verrucaria nigrescens |  | AFTOL-ID 2296 |
|  | EF643807 | Verrucaria polysticta |  | AFTOL-ID 2297 |
|  | EF643813 | Verrucaria tristis |  | AFTOL-ID 2269 |
|  | EF643814 | Verrucaria viridula |  | AFTOL_ID 2299 |
|  | EF643812 | Verrucaria weddellii |  | AFTOL-ID 2300 |
|  | EF643816 | Verrucula arnoldaria |  | AFTOL-ID 2302 |
|  | EF643815 | Verrucula biatorinaria |  | AFTOL-ID 2301 |
|  | EF643817 | Verrucula granulosaria |  | AFTOL-ID 2303 |
|  | EF643819 | Verrucula inconnexaria |  | AFTOL-ID 2305 |
|  | EF643822 | Verruculopsis poeltiana |  | AFTOL-ID 2298 |
|  | DQ470945 | Verticillium dahliae | ATCC 16535 | AFTOL-ID 237 |
|  | FJ176874 | Vibrissea truncorum |  | AFTOL-ID 1322 |
|  | DQ912341 | Vulpicida pinastri |  | AFTOL-ID 198 |
|  | EF643802 | Wahlenbergiella mucosa |  | AFTOL-ID 2264 |
|  | EF643810 | Wahlenbergiella striatula |  | AFTOL-ID 2267 |
|  | AY584641 | Xanthoparmelia conspersa |  | AFTOL-ID 4 |
|  | DQ912352 | Xanthoria elegans |  | AFTOL-ID 214 |
|  | DQ912351 | Xanthoria polycarpa |  | AFTOL-ID 200 |
|  | AY544676 | Xylaria acuta | ATCC 56487 | AFTOL-ID 63 |
|  | AY544648 | Xylaria hypoxylon | spat 03-03 | AFTOL-ID 51 |
|  | FJ176885 | Zygoascus steatolyticus |  | AFTOL-ID 1412 |
| Basidiomycota | | | | |
|  | AY634278 | Agaricostilbum hyphaenes | CBS7811 | AFTOL-ID 675 |
|  | AY635775 | Agaricus bisporus | RWK1885 | AFTOL-ID 448 |
|  | DQ457663 | Agrocybe erebia |  | AFTOL-ID 1807 |
|  | DQ110872 | Agrocybe pediades | PBM2080 | AFTOL-ID 1493 |
|  | AY646101 | Agrocybe praecox | PBM 2310-WA | AFTOL-ID 728 |
|  | DQ110873 | Agrocybe smithii | PBM2298 | AFTOL-ID 1494 |
|  | EF551312 | Albatrellus dispansus |  | AFTOL-ID 2000 |
|  | AY684166 | Albatrellus higanensis | Dai46749 | AFTOL-ID 774 |
|  | DQ457657 | Alloclavaria purpurea |  | AFTOL-ID 1736 |
|  | AY631902 | Amanita brunnescens | PBM2429 | AFTOL-ID 673 |
|  | AY639881 | Ampulloclitocybe clavipes | PBM2474 | AFTOL-ID 542 |
|  | AY575919 | Anamika angustilamellata |  | AFTOL-ID 543 |
|  | AY745709 | Anthracophyllum archeri | PBM2201 | AFTOL-ID 973 |
|  | AY700194 | Armillaria mellea | PBM2470 | AFTOL-ID 449 |
|  | DQ645514 | Asterotremella humicola | PYCC3387T | AFTOL-ID 1552 |
|  | AY635773 | Athelia rolfsii | CBS745.84 | AFTOL-ID 664 |
|  | AY700189 | Aureoboletus thibetanus | HKAS41151 | AFTOL-ID 450 |
|  | DQ419920 | Auriculoscypha anacardiicola |  | AFTOL-ID 1885 |
|  | DQ911614 | Auriscalpium vulgare |  | AFTOL-ID 1897 |
|  | DQ457648 | Baeospora myosura |  | AFTOL-ID 1799 |
|  | AY700184 | Basidioradulum radula | GEL2493 | AFTOL-ID 451 |
|  | AY745730 | Bensingtonia ciliata | CBS7514 | AFTOL-ID 841 |
|  | DQ631903 | Bensingtonia musae | CBS 7965 | AFTOL-ID 1763 |
|  | AY745727 | Bensingtonia yuccicola | CBS7331 | AFTOL-ID 857 |
|  | AY691807 | Bolbitius vitellinus | MTS5020 | AFTOL-ID 730 |
|  | AY684158 | Boletellus projectellus | MB03-118 | AFTOL-ID 713 |
|  | AY647211 | Boletellus shichianus | HKAS43373 nLSU | AFTOL-ID 532 |
|  | AY684153 | Boletinellus merulioides | MB02-199 | AFTOL-ID 575 |
|  | DQ154112 | Boletopsis leucomelaena | PBM2678 | AFTOL-ID 1527 |
|  | DQ234539 | Bondarzewia montana | DAOM 415 | AFTOL-ID 452 |
|  | HM536050 | Boreostereum radiatum |  | RLG-9717-Sp |
|  | DQ089013 | Botryobasidium botryosum | FCUG 1750 | AFTOL-ID 604 |
|  | AY647212 | Botryobasidium subcoronatum | FCUG1286 SWE | AFTOL-ID 614 |
|  | AY745702 | Callistosporium graminicolor |  | AFTOL-ID 978 |
|  | AY701526 | Calocera cornea | GEL5359 | AFTOL-ID 438 |
|  | AY645054 | Calostoma cinnabarinum | AW136MA | AFTOL-ID 439 |
|  | DQ457679 | Camarophyllopsis hymenocephala |  | AFTOL-ID 1892 |
|  | DQ457651 | Camarophyllus basidiosus |  | AFTOL-ID 1759 |
|  | AY700201 | Camarophyllus borealis | PBM2490 | AFTOL-ID 472 |
|  | DQ457652 | Camarophyllus canescens |  | AFTOL-ID 1800 |
|  | AY745708 | Cantharellus cibarius | AW155 | AFTOL-ID 971 |
|  | DQ234540 | Cantharocybe gruberi |  | AFTOL-ID 1017 |
|  | DQ089012 | Catathelasma ventricosum | PBM2403 | AFTOL-ID 1488 |
|  | DQ457653 | Chamaeota sinica |  | AFTOL-ID 1382 |
|  | DQ457654 | Cheimonophyllum candidissimum |  | AFTOL-ID 1765 |
|  | DQ631905 | Chionosphaera apobasidialis | CBS 7430 | AFTOL-ID 1762 |
|  | AY700187 | Chlorophyllum agaricoides | RWK.Sa1 | AFTOL-ID 440 |
|  | DQ457655 | Chromosera cyanophylla |  | AFTOL-ID 1684 |
|  | DQ457656 | Chrysomphalina chrysophylla |  | AFTOL-ID 1523 |
|  | AY700192 | Chrysomyxa arctostaphyli | CFB22246 | AFTOL-ID 442 |
|  | DQ631906 | Cintractia axicola | MP3490 (DNA527) | AFTOL-ID 1920 |
|  | DQ645506 | Cintractia limitata | HAJB 10488; DNA375 | AFTOL-ID 446 |
|  | AY745726 | Cintractia sorghi-vulgaris | CBS104.17 | AFTOL-ID 867 |
|  | AY745693 | Clavaria inaequalis | MB 04-016 | AFTOL-ID 984 |
|  | AY639882 | Clavaria zollingeri | TENN58652 | AFTOL-ID 563 |
|  | AY647208 | Cleistocybe vernalis | PBM1856 WA | AFTOL-ID 721 |
|  | AY684165 | Climacodon septentrionalis | ZWsn | AFTOL-ID 767 |
|  | AY645055 | Clitocybe candicans | PBM2476 MA | AFTOL-ID 541 |
|  | DQ457658 | Clitocybe nebularis |  | AFTOL-ID 1495 |
|  | AY691889 | Clitocybe subditopoda | PBM2489 | AFTOL-ID 533 |
|  | DQ457659 | Clitocybula atroalba |  | AFTOL-ID 1529 |
|  | DQ151452 | Clitocybula oculus | PBM1156 | AFTOL-ID 1554 |
|  | AY700181 | Clitopilus prunulus | TJB6838 | AFTOL-ID 522 |
|  | AY629313 | Colacogloea peniophorae | CBS 684.93 | AFTOL-ID 709 |
|  | AY639884 | Collybia tuberosa | TENN53540 | AFTOL-ID 557 |
|  | DQ457660 | Conocybe apala |  | AFTOL-ID 1675 |
|  | DQ457661 | Coprinopsis atramentaria |  | AFTOL-ID 1496 |
|  | AY635772 | Coprinus comatus | ECV3198 | AFTOL-ID 626 |
|  | EF537893 | Corticium roseum | CBS 205.91 | AFTOL-ID 1943 |
|  | AY684152 | Cortinarius aurilicis | TSJ1998-101 | AFTOL-ID 812 |
|  | AY702013 | Cortinarius iodes | PBM2426 | AFTOL-ID 285 |
|  | AY684151 | Cortinarius sodagnitus | TF2001-094 | AFTOL-ID 811 |
|  | DQ457662 | Cortinarius violaceus |  | AFTOL-ID 814 |
|  | AY700188 | Craterellus fallax | PBM2427 | AFTOL-ID 286 |
|  | DQ645512 | Cryptococcus gastricus | CBS 8636 | AFTOL-ID 1887 |
|  | DQ234541 | Cylindrobasidium laeve | HHB8633-T | AFTOL-ID 453 |
|  | AY635771 | Cyphella digitalis | CBS679.82 | AFTOL-ID 663 |
|  | AY745705 | Cyphellostereum laeve | PBM1662 | AFTOL-ID 982 |
|  | DQ154108 | Cystoderma amianthinum | JFA12566 | AFTOL-ID 1553 |
|  | DQ645521 | Cystofilobasidium capitatum | CBS 6358 | AFTOL-ID 1886 |
|  | DQ645523 | Cystofilobasidium infirmominiatum | CBS 323 | AFTOL-ID 1888 |
|  | AY701525 | Dacryopinax spathularia | GEL5052 | AFTOL-ID 454 |
|  | DQ457664 | Descolea maculata |  | AFTOL-ID 1521 |
|  | DQ917661 | Dictyonema glabratum |  | AFTOL-ID 1995 |
|  | HM536052 | Donkioporia expansa |  | P-188 |
|  | AY700193 | Endocronartium harknessii | CFB22250 | AFTOL-ID 456 |
|  | AY700180 | Entoloma prunuloides | TJB4765 | AFTOL-ID 523 |
|  | AY691891 | Entoloma sinuatum | TJB5349 | AFTOL-ID 524 |
|  | DQ645528 | Entyloma arnoseridis | CBS 203.36 | AFTOL-ID 1801 |
|  | DQ663687 | Entyloma calendulae | CBS 513.93 | AFTOL-ID 1821 |
|  | DQ457665 | Epithele typhae | CBS 203.58 | AFTOL-ID 1724 |
|  | DQ663696 | Erythrobasidium hasegawianum | IAM 12911 | AFTOL-ID 1771 |
|  | AY645056 | Exidia uvapsassa | TA s.n. JAPAN | AFTOL-ID 461 |
|  | AY885162 | Exidiopsis calcea | CBS463.62 | AFTOL-ID 1131 |
|  | AY885167 | Exidiopsis grisea | CBS326.66 | AFTOL-ID 1128 |
|  | DQ663699 | Exobasidium gracile | DSM 4460 | AFTOL-ID 1643 |
|  | DQ667151 | Exobasidium rhododendri | CBS 101457 | AFTOL-ID 1851 |
|  | AY700202 | Fibricium rude | GEL2121 | AFTOL-ID 464 |
|  | EF551315 | Fibulobasidium inconspicuum | CBS 7679 | AFTOL-ID 1956 |
|  | DQ457666 | Flammula alnicola |  | AFTOL-ID 1501 |
|  | AY639883 | Flammulina velutipes | TENN52002 | AFTOL-ID 558 |
|  | AY684157 | Fomitiporia mediterranea | 3/22-7 | AFTOL-ID 688 |
|  | AY684164 | Fomitopsis pinicola | MB03-036 | AFTOL-ID 770 |
|  | AY885166 | Fuscoporia viticola | PBM 2377 | AFTOL-ID 977 |
|  | DQ457668 | Galerina atkinsoniana |  | AFTOL-ID 1760 |
|  | DQ457669 | Galerina marginata |  | AFTOL-ID 465 |
|  | AY684163 | Ganoderma tsugae | ZWsn | AFTOL-ID 771 |
|  | DQ785787 | Glaciozyma antarctica | PYCC5541T | AFTOL-ID 1550 |
|  | DQ154109 | Gliophorus laetus | PBM2280 | AFTOL-ID 1555 |
|  | HM536054 | Gloeophyllum carbonarium |  | FP-97972 |
|  | HM536056 | Gloeophyllum mexicanum |  | FP-104133-Sp |
|  | HM536058 | Gloeophyllum odoratum |  | CBS-444.61 |
|  | HM536059 | Gloeophyllum protractum |  | H80 |
|  | HM536061 | Gloeophyllum sepiarium |  | Wilcox-3BB |
|  | HM536063 | Gloeophyllum striatum |  |  |
|  | HM536065 | Gloeophyllum subferrugineum |  | FPRI-508 |
|  | HM536067 | Gloeophyllum trabeum |  | 1320 |
|  | DQ097343 | Gloiocephala aquatica | CIEFAP50 | AFTOL-ID 517 |
|  | DQ534669 | Gomphidius roseus | MB 95-038; Gro1 | AFTOL-ID 1780 |
|  | AY647207 | Gomphus clavatus | G071 | AFTOL-ID 725 |
|  | AY745729 | Granulobasidium vellereum | CBS52.84 | AFTOL-ID 887 |
|  | AY629318 | Grifola frondosa | DSH s.n. Mass | AFTOL-ID 701 |
|  | AY645050 | Grifola sordulenta | TENN55054 | AFTOL-ID 562 |
|  | EF551318 | Guehomyces pullulans | CBS 2532 | AFTOL-ID 1958 |
|  | AY745711 | Guepiniopsis buccina | PBM2264 | AFTOL-ID 888 |
|  | DQ521421 | Gymnoconia peckiana |  | AFTOL-ID 1630 |
|  | AY700186 | Gymnopilus spectabilis | PBM2471 | AFTOL-ID 467 |
|  | DQ457670 | Gymnopus contrarius |  | AFTOL-ID 1758 |
|  | AY640619 | Gymnopus dryophilus | TENN57012 | AFTOL-ID 559 |
|  | AY629316 | Gymnosporangium juniperi-virginianae | PBM2530 | AFTOL-ID 712 |
|  | DQ521414 | Haplotrichum conspersum |  | AFTOL-ID 1766 |
|  | AY745703 | Hebeloma velutipes | PBM2277 | AFTOL-ID 980 |
|  | AY885168 | Helicobasidium longisporum | CBS324.47 | AFTOL-ID 1160 |
|  | HM536069 | Heliocybe sulcata |  |  |
|  | DQ457671 | Hemimycena gracilis |  | AFTOL-ID 1732 |
|  | DQ411538 | Hericium americanum | PBM2498 | AFTOL-ID 469 |
|  | AY631900 | Hydnellum geogenium | PBM2382 | AFTOL-ID 680 |
|  | AY635770 | Hydnochaete duportii | CBS939.96 | AFTOL-ID 666 |
|  | AY700199 | Hydnum albomagnum | PBM2512 | AFTOL-ID 471 |
|  | DQ457674 | Hydropus marginellus |  | AFTOL-ID 1720 |
|  | EF551314 | Hygroaster albellus |  | AFTOL-ID 1997 |
|  | EF561625 | Hygroaster nodulisporus |  | AFTOL-ID 2020 |
|  | DQ457675 | Hygrocybe cantharellus |  | AFTOL-ID 1714 |
|  | DQ457676 | Hygrocybe coccinea |  | AFTOL-ID 1715 |
|  | DQ457677 | Hygrocybe miniata f. longipes |  | AFTOL-ID 1891 |
|  | AY684156 | Hygrophoropsis aurantiaca | MB03-127 | AFTOL-ID 714 |
|  | DQ457672 | Hygrophorus auratocephalus |  | AFTOL-ID 1727 |
|  | AY635769 | Hygrophorus flavodiscus | PBM2509 | AFTOL-ID 641 |
|  | DQ457678 | Hygrophorus pudorinus |  | AFTOL-ID 1723 |
|  | AY691890 | Hymenopellis furfuracea | PBM2440 | AFTOL-ID 538 |
|  | AY645051 | Hymenopellis radicata | TENN59235 | AFTOL-ID 561 |
|  | AY646100 | Hyphodontia gossypina | GEL 5042 | AFTOL-ID 599 |
|  | AY635774 | Hypholoma sublateritium | JS031107 | AFTOL-ID 597 |
|  | DQ917664 | Hypsizygus tessulatus |  | AFTOL-ID 1898 |
|  | DQ457682 | Infundibulicybe gibba |  | AFTOL-ID 1508 |
|  | AY702014 | Inocybe cookei | PBM2459 | AFTOL-ID 520 |
|  | AY745700 | Inocybe maculata | PBM2446 | AFTOL-ID 476 |
|  | AY646099 | Insolibasidium deformans | DJM 183.1 | AFTOL-ID 722 |
|  | DQ667161 | Itersonilia perplexans | CBS 286.50 | AFTOL-ID 1896 |
|  | DQ911606 | Jahnoporus hirtus |  | AFTOL-ID 1687 |
|  | AY745720 | Kondoa malvinella | CBS6082 | AFTOL-ID 859 |
|  | AY745728 | Kriegeria eriophori | CBS101449 | AFTOL-ID 886 |
|  | AY745696 | Kuehneola uredinis | PBM2577 | AFTOL-ID 987 |
|  | DQ457684 | Kuehneromyces rostratus |  | AFTOL-ID 1676 |
|  | AY700200 | Laccaria ochropurpurea | PBM2443 | AFTOL-ID 477 |
|  | DQ097347 | Lachnella villosa | CBS609.87 | AFTOL-ID 525 |
|  | AY700198 | Lacrymaria velutina | PBM2439 | AFTOL-ID 478 |
|  | AY631899 | Lactarius deceptivus | PBM2462 | AFTOL-ID 682 |
|  | AY631898 | Lactarius lignyotus | PBM2424 | AFTOL-ID 681 |
|  | AY684162 | Laetiporus sulphureus | ZWsn | AFTOL-ID 769 |
|  | DQ457685 | Lepiota cristata |  | AFTOL-ID 1625 |
|  | DQ234538 | Lepista irina |  | AFTOL-ID 815 |
|  | DQ911601 | Leucoagaricus barssii |  | AFTOL-ID 1899 |
|  | AY646098 | Leucosporidium scottii | CBS 614 | AFTOL-ID 718 |
|  | DQ094787 | Macrocystidia cucumis | HKAS 31464 | AFTOL-ID 1343 |
|  | DQ411537 | Macrolepiota dolichaula | HKAS38718 | AFTOL-ID 529 |
|  | AY745725 | Malassezia furfur | CBS1878 | AFTOL-ID 855 |
|  | AY745724 | Malassezia pachydermatis | CBS1879 | AFTOL-ID 856 |
|  | AY700196 | Mallocybe dulcamara | JV19652F | AFTOL-ID 482 |
|  | AY635776 | Marasmius alliaceus | TENN55620 | AFTOL-ID 556 |
|  | DQ156126 | Marasmius oreades | PBM2701 | AFTOL-ID 1525 |
|  | DQ457686 | Marasmius rotula |  | AFTOL-ID 1505 |
|  | AY702016 | Megacollybia platyphylla | PBM2431 | AFTOL-ID 534 |
|  | AY635778 | Megacollybia platyphylla | TENN59432 | AFTOL-ID 560 |
|  | DQ457687 | Melanoleuca verrucipes |  | AFTOL-ID 818 |
|  | DQ789979 | Melanotaenium endogenum | CBS 481.91 | AFTOL-ID 1918 |
|  | EF561635 | Mesophellia glauca |  | AFTOL-ID 1683 |
|  | DQ789982 | Microbotryum violaceum | CBS 438.34 | AFTOL-ID 1819 |
|  | DQ831009 | Mixia osmundae | IAM14324 | AFTOL-ID 1773 |
|  | DQ831011 | Moesziomyces bullatus | CBS 425.34 | AFTOL-ID 1820 |
|  | DQ831016 | Mrakia frigida | CBS 5266 | AFTOL-ID 1818 |
|  | AY885163 | Multiclavula mucida | CBS277.94 | AFTOL-ID 1130 |
|  | DQ457691 | Mycena amabilissima |  | AFTOL-ID 1686 |
|  | DQ457692 | Mycena amicta |  | AFTOL-ID 1908 |
|  | DQ470811 | Mycena aurantiidisca |  | AFTOL-ID 1685 |
|  | DQ470812 | Mycena auricoma |  | AFTOL-ID 1341 |
|  | AY647216 | Mycena galericulata | PBM2407 WA | AFTOL-ID 727 |
|  | DQ470813 | Mycena plumbea |  | AFTOL-ID 1631 |
|  | DQ911597 | Mycocalia denudata | CBS 494.85 | AFTOL-ID 2018 |
|  | AY745707 | Mythicomyces corneipes | PBM1210 | AFTOL-ID 972 |
|  | DQ831020 | Naohidea sebacea | CBS 8477 | AFTOL-ID 1761 |
|  | DQ457681 | Nematoloma longisporum |  | AFTOL-ID 1893 |
|  | DQ470814 | Neohygrophorus angelesianus |  | AFTOL-ID 1719 |
|  | HM536071 | Neolentinus adhaerens |  |  |
|  | HM536073 | Neolentinus kauffmanii |  |  |
|  | HM536077 | Neolentinus lepideus |  |  |
|  | DQ986295 | Nidula niveotomentosa |  | AFTOL-ID 1945 |
|  | EF535276 | Nidularia farcta | CBS 156.42 | AFTOL-ID 1933 |
|  | DQ470815 | Nivatogastrium nubigenum |  | AFTOL-ID 1500 |
|  | AY745723 | Occultifur externus | CBS8732 | AFTOL-ID 860 |
|  | DQ470816 | Omphalotus olearius |  | AFTOL-ID 1718 |
|  | DQ470817 | Panaeolus sphinctrinus |  | AFTOL-ID 1499 |
|  | AY645059 | Paxillus vernalis | MB062_CHINA | AFTOL-ID 715 |
|  | DQ094786 | Peniophora cinerea | CBS 404.74 | AFTOL-ID 1491 |
|  | AY700185 | Peniophorella praetermissa | GEL2182 | AFTOL-ID 518 |
|  | AY629319 | Phaeolus schweinitzii | Andy Wilson s.n. NY | AFTOL-ID 702 |
|  | AY885165 | Phallus hadriani | KH11092003.1 | AFTOL-ID 683 |
|  | DQ831021 | Phleogena faginea |  | AFTOL-ID 1889 |
|  | DQ470818 | Pholiota squarrosa |  | AFTOL-ID 1627 |
|  | DQ470819 | Pholiotina filaris |  | AFTOL-ID 1498 |
|  | DQ097349 | Physalacria bambusae | CBS712.83 | AFTOL-ID 515 |
|  | AY745698 | Pileolaria toxicodendri | PBM2579 | AFTOL-ID 988 |
|  | AY629314 | Platygloea disciformis | IFO32431 | AFTOL-ID 710 |
|  | EF537894 | Pleurocybella porrigens |  | AFTOL-ID 2001 |
|  | AY645052 | Pleurotus ostreatus | TENN53662 | AFTOL-ID 564 |
|  | DQ470820 | Plicaturopsis crispa |  | AFTOL-ID 1924 |
|  | DQ094788 | Pluteus atromarginatus | HKAS 31573 | AFTOL-ID 1340 |
|  | AY634279 | Pluteus romellii | ECV3201 | AFTOL-ID 625 |
|  | EF537892 | Podoscypha petalodes subsp. rosulata | CBS 659.84 | AFTOL-ID 1931 |
|  | DQ470821 | Podoserpula pusio |  | AFTOL-ID 1522 |
|  | AY634276 | Polyozellus multiplex | BK290899 | AFTOL-ID 679 |
|  | AY634275 | Polyozellus multiplex | PBM2412 | AFTOL-ID 677 |
|  | AY629320 | Polyporus squamosus | Andy Wilson s.n. Mass | AFTOL-ID 704 |
|  | DQ457673 | Porotheleum fimbriatum | CBS 788.86 | AFTOL-ID 1725 |
|  | DQ534643 | Porphyrellus porphyrosporus | MB97-023 | AFTOL-ID 1779 |
|  | DQ354555 | Prospodium lippiae | U152 | AFTOL-ID 1401 |
|  | DQ110874 | Psathyrella candolleana | JCS0804A | AFTOL-ID 1507 |
|  | AY645058 | Psathyrella rhodospora | MP133 MN | AFTOL-ID 723 |
|  | DQ470822 | Psathyrella spadicea |  | AFTOL-ID 1628 |
|  | DQ154111 | Pseudoarmillariella ectypoides | PBM1588 | AFTOL-ID 1557 |
|  | EF551313 | Pseudoclitocybe cyathiformis |  | AFTOL-ID 1998 |
|  | DQ831025 | Pseudohydnum gelatinosum |  | AFTOL-ID 1633 |
|  | AY745712 | Pseudozyma flocculosa | CBS102.71 | AFTOL-ID 864 |
|  | DQ470823 | Psilocybe montana |  | AFTOL-ID 820 |
|  | AY629315 | Pterula echo | DJM302S58 | AFTOL-ID 711 |
|  | DQ415277 | Puccinia arundinariae | MCA2494 | AFTOL-ID 1404 |
|  | DQ354527 | Puccinia hordei | MCA2391 | AFTOL-ID 1402 |
|  | EF561641 | Puccinia malvacearum |  | AFTOL-ID 1629 |
|  | DQ831028 | Puccinia poarum |  | AFTOL-ID 1027 |
|  | AY745697 | Pucciniastrum circaeae | PBM2576 | AFTOL-ID 985 |
|  | AY645057 | Ramaria rubella | PBM2408 WA | AFTOL-ID 724 |
|  | AY700183 | Resinicium bicolor | GEL4664 | AFTOL-ID 519 |
|  | DQ831032 | Rhamphospora nymphaeae | CBS 72.38 | AFTOL-ID 1645 |
|  | AY639880 | Rhodocollybia maculata | PBM2481 | AFTOL-ID 540 |
|  | AY700182 | Rhodocybe mundula | TJB7599 | AFTOL-ID 521 |
|  | AY745719 | Rhodosporidium fluviale | CBS6568 | AFTOL-ID 853 |
|  | DQ832191 | Rhodosporidium toruloides | PYCC4416 | AFTOL-ID 1547 |
|  | AY646097 | Rhodotorula glutinis | CBS 20 | AFTOL-ID 720 |
|  | DQ832196 | Rhodotorula hinnulea | CBS 8079 | AFTOL-ID 1764 |
|  | AY631901 | Rhodotorula hordea | CBS6403 | AFTOL-ID 674 |
|  | DQ832198 | Rhodotorula mucilaginosa | PYCC5166 | AFTOL-ID 1548 |
|  | EF551316 | Rhodotorula nothofagi | CBS 8166 | AFTOL-ID 1935 |
|  | AY700195 | Rickenella fibula | PBM2503 | AFTOL-ID 486 |
|  | DQ832205 | Sakaguchia dacryoidea | PYCC4491 | AFTOL-ID 1551 |
|  | AY691887 | Sarcomyxa serotina | PBM2519 | AFTOL-ID 536 |
|  | DQ832210 | Schizonella melanogramma | CBS 174.42 | AFTOL-ID 1722 |
|  | AY571023 | Schizophyllum radiatum | CBS 301.32 | AFTOL-ID 516 |
|  | DQ521406 | Sebacina incrustans |  | AFTOL-ID 1626 |
|  | AY745706 | Simocybe serrulata | PBM2536 | AFTOL-ID 970 |
|  | AY647214 | Sistotrema confluens | FCUG298 SWE | AFTOL-ID 613 |
|  | DQ457641 | Sistotrema coronilla | FCUG 863 | AFTOL-ID 618 |
|  | AY647210 | Sistotrema oblongisporum | FCUG2422 SWE | AFTOL-ID 617 |
|  | AY647213 | Sistotrema raduloides | FCUG613 SWE | AFTOL-ID 619 |
|  | AY647215 | Sistotrema sernanderi | FCUG1049 SWE | AFTOL-ID 620 |
|  | AY629321 | Sparassis crispa | HKAS43721 | AFTOL-ID 703 |
|  | DQ354521 | Sphenospora kevorkianii | U10 | AFTOL-ID 1399 |
|  | AY629322 | Spongipellis pachyodon | ZWsn RI | AFTOL-ID 705 |
|  | AY745718 | Sporidiobolus johnsonii | CBS5470 | AFTOL-ID 854 |
|  | DQ832228 | Sporisorium reilianum | MP3000 | AFTOL-ID 490 |
|  | EF537895 | Sporobolomyces griseoflavus | CBS 7284 | AFTOL-ID 1938 |
|  | DQ832234 | Sporobolomyces roseus | PYCC4463 | AFTOL-ID 1549 |
|  | AY745717 | Sporobolomyces subbrunneus | CBS7196 | AFTOL-ID 858 |
|  | AY745716 | Sterigmatomyces halophilus | CBS4609 | AFTOL-ID 863 |
|  | AY645053 | Strobilomyces floccopus | PBM2436 | AFTOL-ID 565 |
|  | AY684155 | Strobilomyces floccopus | MB03-102 | AFTOL-ID 716 |
|  | AY646102 | Stropharia ambigua | PBM 2257 | AFTOL-ID 726 |
|  | AY684154 | Suillus pictus | MB03-002 | AFTOL-ID 717 |
|  | DQ832238 | Sympodiomycopsis paphiopedili | IAM13459 | AFTOL-ID 1772 |
|  | DQ917658 | Thanatephorus cucumeris | CBS 253.29 | AFTOL-ID 2022 |
|  | DQ832241 | Thecaphora spilanthis | JAG 53 | AFTOL-ID 1913 |
|  | DQ832244 | Tilletia controversa | MP2525 | AFTOL-ID 493 |
|  | AY745715 | Tilletiaria anomala | CBS436.72 | AFTOL-ID 865 |
|  | AY745713 | Tilletiopsis minor | CBS543.5 | AFTOL-ID 866 |
|  | AY745714 | Tilletiopsis washingtonensis | CBS544.5 | AFTOL-ID 868 |
|  | AY684159 | Trametes versicolor | MB73 | AFTOL-ID 768 |
|  | AY635768 | Trechispora alnicola | CBS577.83 | AFTOL-ID 665 |
|  | EF561626 | Tremella aurantia |  | AFTOL-ID 1520 |
|  | DQ156127 | Tremella aurantia | E6123 | AFTOL-ID 1519 |
|  | EF551317 | Tremella globispora | CBS 6972 | AFTOL-ID 1959 |
|  | AY700197 | Tricholoma aestuans | PBM2494 | AFTOL-ID 497 |
|  | AY647209 | Tricholoma saponaceum | PBM2514 MA | AFTOL-ID 672 |
|  | AY691888 | Tricholomopsis decora | PBM2482 | AFTOL-ID 537 |
|  | DQ836002 | Trichosporon lignicola | CBS 219.34 | AFTOL-ID 1802 |
|  | EF537891 | Trichosporon ovoides | CBS 7556 | AFTOL-ID 1928 |
|  | AY700190 | Tubaria confragosa | PBM2105 | AFTOL-ID 498 |
|  | DQ156128 | Tubaria serrulata | E8069 | AFTOL-ID 1528 |
|  | DQ536415 | Tubaria vinicolor |  | AFTOL-ID 499 |
|  | DQ836005 | Udeniomyces puniceus | CBS 5689 | AFTOL-ID 1822 |
|  | DQ838576 | Urocystis colchici | CBS 283.28 | AFTOL-ID 1647 |
|  | AY745704 | Uromyces appendiculatus | Ua39 | AFTOL-ID 976 |
|  | AY745695 | Uromyces viciae-fabae | PBM2573 | AFTOL-ID 986 |
|  | DQ846888 | Ustanciosporium standleyanum | JAG 73 | AFTOL-ID 1915 |
|  | DQ094784 | Ustilago tritici | CBS 669.70 | AFTOL-ID 1398 |
|  | HM536079 | Veluticeps abietina |  | GB-398 |
|  | HM536081 | Veluticeps berkeleyi |  | HHB-8594-Sp |
|  | HM536083 | Veluticeps fimbriata |  | L-10628-Sp |
|  | DQ470825 | Verrucospora flavofusca |  | AFTOL-ID 655 |
|  | AY745710 | Volvariella gloiocephala | PBM2272 | AFTOL-ID 890 |
|  | AY885164 | Waitea circinata | CBS315.84 | AFTOL-ID 1129 |
|  | DQ847516 | Wallemia ichthyophaga | EXF1059 | AFTOL-ID 1901 |
|  | DQ847517 | Wallemia muriae | EXF1054 | AFTOL-ID 1909 |
|  | DQ847518 | Wallemia sebi | EXF483 | AFTOL-ID 1910 |
|  | DQ470826 | Xeromphalina campanella |  | AFTOL-ID 1524 |
| Chytridiomycota | | | | |
|  | AY546693 | Batrachochytrium dendrobatidis | JEL197 | AFTOL-ID 21 |
|  | DQ273823 | Boothiomyces macroporosum | PL AUS 21 (U. Alabama) | AFTOL-ID 689 |
|  | DQ273836 | Chytriomyces hyalinus | MP4 (U. Alabama) | AFTOL-ID 1537 |
|  | DQ273839 | Chytriomyces spinosus | JEL59 (J. E. Longcore) | AFTOL-ID 1540 |
|  | AY546688 | Cladochytrium replicatum | JEL180 | AFTOL-ID 27 |
|  | DQ273784 | Entophlyctis helioformis | JEL326 (J. E. Longcore) | AFTOL-ID 40 |
|  | DQ273778 | Gaertneriomyces semiglobifer | UBC 91-10 (Berkeley Microgarden) | AFTOL-ID 34 |
|  | DQ273771 | Hyaloraphidium curvatum | SAG 235-1 | AFTOL-ID 26 |
|  | DQ273824 | Kappamyces laurelensis | PL 98 (U. Alabama) | AFTOL-ID 690 |
|  | DQ273815 | Lobulomyces angularis | JEL45 (J. E. Longcore) | AFTOL-ID 630 |
|  | DQ273777 | Monoblepharella mexicana | UCB 78-1 (Berkeley Microgarden) | AFTOL-ID 33 |
|  | DQ273813 | Phlyctochytrium planicorne | JEL47 (J. E. Longcore) | AFTOL-ID 628 |
|  | DQ273838 | Podochytrium dentatum | JEL30 (J. E. Longcore) | AFTOL-ID 1539 |
|  | AY546686 | Polychytrium aggregatum | JEL109 | AFTOL-ID 24 |
|  | DQ273775 | Rhizophlyctis harderi | JEL171 (J. E. Longcore) | AFTOL-ID 31 |
|  | DQ273787 | Rhizophlyctis rosea | JEL318 (J. E. Longcore) | AFTOL-ID 43 |
|  | DQ273770 | Rhizophydium brooksianum | JEL136 (J. E. Longcore) | AFTOL-ID 22 |
|  | DQ273781 | Rhizophydium sphaerotheca | JEL299 (J. E. Longcore) | AFTOL-ID 37 |
|  | AY546692 | Spizellomyces punctatus | ATCC 48900 | AFTOL-ID 182 |
|  | DQ273819 | Synchytrium decipiens |  | AFTOL-ID 634 |
|  | DQ273820 | Synchytrium macrosporum |  | AFTOL-ID 635 |
|  | DQ273826 | Triparticalcar arcticum | Canadian Collection of Fungus Cultures #BR 59 | AFTOL-ID 696 |
| Kickxellomycotina | | | | |
|  | AY546689 | Coemansia reversa | NRRL1564 | AFTOL-ID 140 |
|  | DQ273791 | Dimargaris bacillispora | NRRL 2808 | AFTOL-ID 136 |
|  | DQ273809 | Furculomyces boomerangus | AUS-42-7 (U. Kansas) | AFTOL-ID 303 |
|  | DQ273830 | Orphella haysii | NS-35-W16 (U. Kansas) | AFTOL-ID 1062 |
|  | DQ273773 | Smittium culisetae | Col-18-3 (U. Kansas) | AFTOL-ID 29 |
|  | DQ273801 | Spiromyces aspiralis | NRRL 22631 | AFTOL-ID 185 |
|  | DQ273810 | Spiromyces minutus | NRRL 3067 | AFTOL-ID 327 |
| Mucoromycotina | | | | |
|  | DQ273812 | Cokeromyces recurvatus | NRRL 2808-h | AFTOL-ID 627 |
|  | DQ273788 | Endogone lactiflua |  | AFTOL-ID 45 |
|  | DQ273811 | Endogone pisiformis | Canadian Collection of Fungus Cultures #DAOM 233144 | AFTOL-ID 539 |
|  | DQ273794 | Mortierella verticillata | NRRL 6337 | AFTOL-ID 141 |
|  | DQ273800 | Phycomyces blakesleeanus | NRRL 1555 | AFTOL-ID 184 |
|  | DQ273817 | Rhizopus stolonifer | Canadian Collection of Fungus Cultures #DAOM 225708 | AFTOL-ID 632 |
|  | DQ273797 | Umbelopsis ramanniana | NRRL 5844 | AFTOL-ID 144 |
| Glomeromycota | | | | |
|  | DQ273790 | Glomus intraradices | Belgian Coordination Collection of Microorganisms #MUCL43194 | AFTOL-ID 48 |
|  | DQ273828 | Glomus intraradices | GINCO #4695rac-11G2 | AFTOL-ID 845 |
|  | DQ273793 | Glomus mosseae | INVAM #UT101 | AFTOL-ID 139 |
|  | DQ273827 | Paraglomus occultum | INVAM #IA702 | AFTOL-ID 844 |
|  | DQ273792 | Scutellospora heterogama | INVAM #FL225 | AFTOL-ID 138 |
| Blastocladiomycota | | | | |
|  | AY552525 | Allomyces arbuscula | Brazil 2 (UC Berkeley Microgarden) | AFTOL-ID 300 |
|  | DQ273808 | Blastocladiella emersonii | UCB 49-1 (Berkeley Microgarden) | AFTOL-ID 302 |
|  | DQ273767 | Coelomomyces stegomyiae |  | AFTOL-ID 18 |
|  | DQ273768 | Physoderma maydis |  | AFTOL-ID 19 |
| Entomophthoromycotina | | | | |
|  | DQ273807 | Basidiobolus ranarum | NRRL 34594 | AFTOL-ID 301 |
|  | AY546691 | Conidiobolus coronatus | NRRL28638 | AFTOL-ID 137 |
|  | DQ273772 | Entomophthora muscae | ARSEF3074 | AFTOL-ID 28 |
| Zoopagomycotina | | | | |
|  | DQ273796 | Kuzuhaea moniliformis | NRRL 13723 | AFTOL-ID 143 |
|  | AY546690 | Piptocephalis corymbifera | NRRL2385 | AFTOL-ID 145 |
|  | DQ273795 | Rhopalomyces elegans | NRRL A-10835 | AFTOL-ID 142 |
| Neocallimastigomycota | | | | |
|  | DQ273829 | Cyllamyces aberensis | EO14 (G. W. Griffith) | AFTOL-ID 846 |
| Olpidiaceae | | | | |
|  | DQ273818 | Olpidium brassicae |  | AFTOL-ID 633 |
| Rozella clade | | | | |
|  | DQ273803 | Rozella allomycis | UCB 47-054 (Berkeley Microgarden) | AFTOL-ID 297 |
