# Supplementary material for: Factors That Affect Large Subunit Ribosomal DNA Amplicon Sequencing Studies of Fungal Communities: Classification Method, Primer Choice, and Error
Source: PLoS One. 2012 Apr 27;7(4):e35749. doi: 10.1371/journal.pone.0035749 (PMC3338786; doi:10.1371/journal.pone.0035749)
Supplement: Table S2 — (DOC) [file pone.0035749.s008.doc]

Table S2: Taxonomic breakdown of the simulated short read large subunit ribosomal DNA set for each primer. Sample sizes for the 5’ and 3’ primers (LR0R and LR7) were smaller than for the internal primers (LR3 and LR5) because the LR0R primer-binding site may have been trimmed from sequences prior to submission, or because sequences were not long enough to span the LR7 primer-binding site.

|  | **Primer** | | | |
| --- | --- | --- | --- | --- |
| **Taxonomic group** | **LR0R** | **LR3** | **LR5** | **LR7** |
| Ascomycota | 17 | 430 | 411 | 1 |
| Basidiomycota | 38 | 320 | 301 | 6 |
| Chytridiomycota | 18 | 21 | 22 | 22 |
| Kickxellomycotina | 2 | 7 | 6 | 5 |
| Mucoromycotina | 5 | 7 | 7 | 7 |
| Glomeromycota | 5 | 5 | 5 | 1 |
| Blastocladiomycota | 2 | 4 | 4 | 4 |
| Entomophthoromycotina | 1 | 3 | 3 | 3 |
| Zoopagomycotina | 1 | 3 | 3 | 3 |
| Neocallimastigomycota | 1 | 1 | 1 | 1 |
| Olpidiaceae | 1 | 1 | 1 | 1 |
| Rozella clade | 1 | 1 | 1 | 1 |
| Total | 92 | 803 | 765 | 55 |
